# Liver regeneration after partial hepatectomy is improved in the absence of aryl hydrocarbon receptor

**DOI:** 10.1038/s41598-022-19733-0

**Published:** 2022-09-14

**Authors:** Claudia M. Rejano-Gordillo, Francisco J. González-Rico, Beatriz Marín-Díaz, Ana Ordiales-Talavero, Ana Nacarino-Palma, Ángel C. Román, Jaime M. Merino, Pedro M. Fernández-Salguero

**Affiliations:** 1 Departamento de Bioquímica y Biología Molecular, Facultad de Ciencias, Universidad de Extremadura, Avenida de Elvas s/n, 06071 Badajoz, Spain; 2 Instituto Universitario de Investigación Biosanitaria de Extremadura (INUBE), Avenida de la Investigación s/n, 06071 Badajoz, Spain

**Keywords:** Biochemistry, Biological techniques, Cell biology, Chemical biology, Genetics, Immunology, Molecular biology, Stem cells, Biomarkers, Gastroenterology

## Abstract

The liver is among the few organs having the ability to self-regenerate in response to a severe damage compromising its functionality. The Aryl hydrocarbon receptor (Ahr) is a transcription factor relevant for the detoxification of xenobiotics but also largely important for liver development and homeostasis. Hence, liver cell differentiation is developmentally modulated by Ahr through the controlled expression of pluripotency and stemness-inducing genes. Here, 2/3 partial hepatectomy (PH) was used as a clinically relevant approach to induce liver regeneration in Ahr-expressing (*Ahr*^+*/*+^) and Ahr-null (*Ahr*^−/−^) mice. Ahr expression and activity were early induced after 2/3 PH to be gradually downmodulated latter during regeneration. *Ahr*^−/−^ mice triggered liver regeneration much faster than *AhR*^+*/*+^ animals, although both reached full regeneration at the latest times. At initial stages after PHx, earlier regenerating *Ahr*^−/−^ livers had upregulation of cell proliferation markers and increased activation of signalling pathways related to stemness such as Hippo-YAP and Wnt/β-catenin, concomitantly with the induction of pro-inflammatory cytokines TNFa, IL6 and p65. These phenotypes, together with the improved metabolic adaptation of *Ahr*^−/−^ mice after PHx and their induced sustained cell proliferation, could likely result from the expansion of undifferentiated stem cells residing in the liver expressing OCT4, SOX2, KLF4 and NANOG. We propose that Ahr needs to be induced early during regeneration to fine-tune liver regrowth to physiological values. Since Ahr deficiency did not result in liver overgrowth, its transient pharmacological inhibition could serve to improve liver regeneration in hepatectomized and transplanted patients and in those exposed to damaging liver toxins and carcinogens.

## Introduction

Under homeostatic conditions, the adult liver has a differentiated, non-proliferative and polyploid phenotype with negligible cell renewal needed for its metabolic, detoxifying and energetic functions^1,2^. Injuries and severe pathological conditions can damage the liver eventually reducing its total mass. However, the liver is among the few organs in the body with an efficient regenerative capacity to recover its size, architecture and function^3,4^.

Two-thirds partial hepatectomy (PHx) in rodents represents a convenient model to investigate the complexity of the signalling pathways critical to control liver regeneration^5^. Other models of liver regeneration use chemical compounds such as carbon tetrachloride (CCl_4_) to cause an acute injury associated to inflammation and tissue necrosis^6^. After PHx, hepatocytes are released from their quiescent state and, together with other hepatic cells, enter cell cycle and start to divide. This proliferative process is in a large part driven by the activation of the immune system with the release of IL6 and TNFα by macrophages^7^. Thus, regeneration induced by PHx is mainly produced by the proliferation of quiescent hepatocytes with a marginal implication of liver stem cells^4,6,8^.

The central network of genes controlling the balance between pluripotency and differentiation is formed by transcription factors OCT4, NANOG and SOX2^9^. These transcription factors maintain an undifferentiated state in the liver similar to that of hepatic oval cells and their positive regulation is related to the development of hepatocarcinoma arising from liver cancer stem cells^10–13^. Moreover, the expression of OCT4 and NANOG is positively regulated in normal and tumor liver cells by Wnt/β-catenin^14^. An additional signalling pathway involved in liver regeneration is mediated by Hippo since ectopic activation of its effector YAP in hepatocytes causes their dedifferentiation and the gaining of progenitor cell properties^15^. The hepatic metabolism is also markedly affected by PHx with a switch in energy source from glycolytic to lipidic metabolism in hepatocytes^16,17^.

The Aryl hydrocarbon receptor (Ahr) has important roles in liver development and homeostasis. Ahr deficient mice (*Ahr*^−/−^) have reduced liver size with variable degrees of fibrosis^18,19^ and a intrahepatic portosystemic shunt not resolved from preweaning to adulthood^19,20^. Recent work from our group has revealed that Ahr is needed for the proper maturation, differentiation and polyploidization that takes place during the transition from an immature (preweaning) to a mature (adult) liver finally resulting in a non-proliferative organ. Accordingly, the *Ahr*^−/−^ liver phenotype includes increased proliferation with deficient polyploidization and upregulation of PI3K/AKT, ERK, and Wnt/β-Cat signalling to mTORC1 and higher levels of mitochondrial oxidative phosphorylation^21^. Furthermore, Ahr serves to adjust the regenerative potential of the liver upon acute toxic injury by controlling the expansion of undifferentiated cells expressing pluripotency markers Oct4 and Nanog^22^.

In this work, we have used a 2/3 PHx approach on *Ahr*^+*/*+^ and *Ahr*^−/−^ livers to further investigate the role of Ahr in liver regeneration and in the mechanisms involved. Our main finding is that Ahr depletion improves liver regeneration in response to a severe organ damage most likely driven by the expansion of hepatic stem cells already known to improve regeneration after acute toxic injury. Signalling through PI3K/AKT, Wnt/β-Cat and Hippo pathways seem to be involved in the regulatory process in an Ahr-dependent manner. These results could open the possibility of pharmacological intervention to modulate Ahr expression to stimulate the recovery of the liver structure and functionality after damage induced by toxins or pathological processes, and even accelerate liver regeneration following liver transplantation.

## Results

### Ahr expression is induced after 2/3 PHx in wild type mice

**Figure 1:**
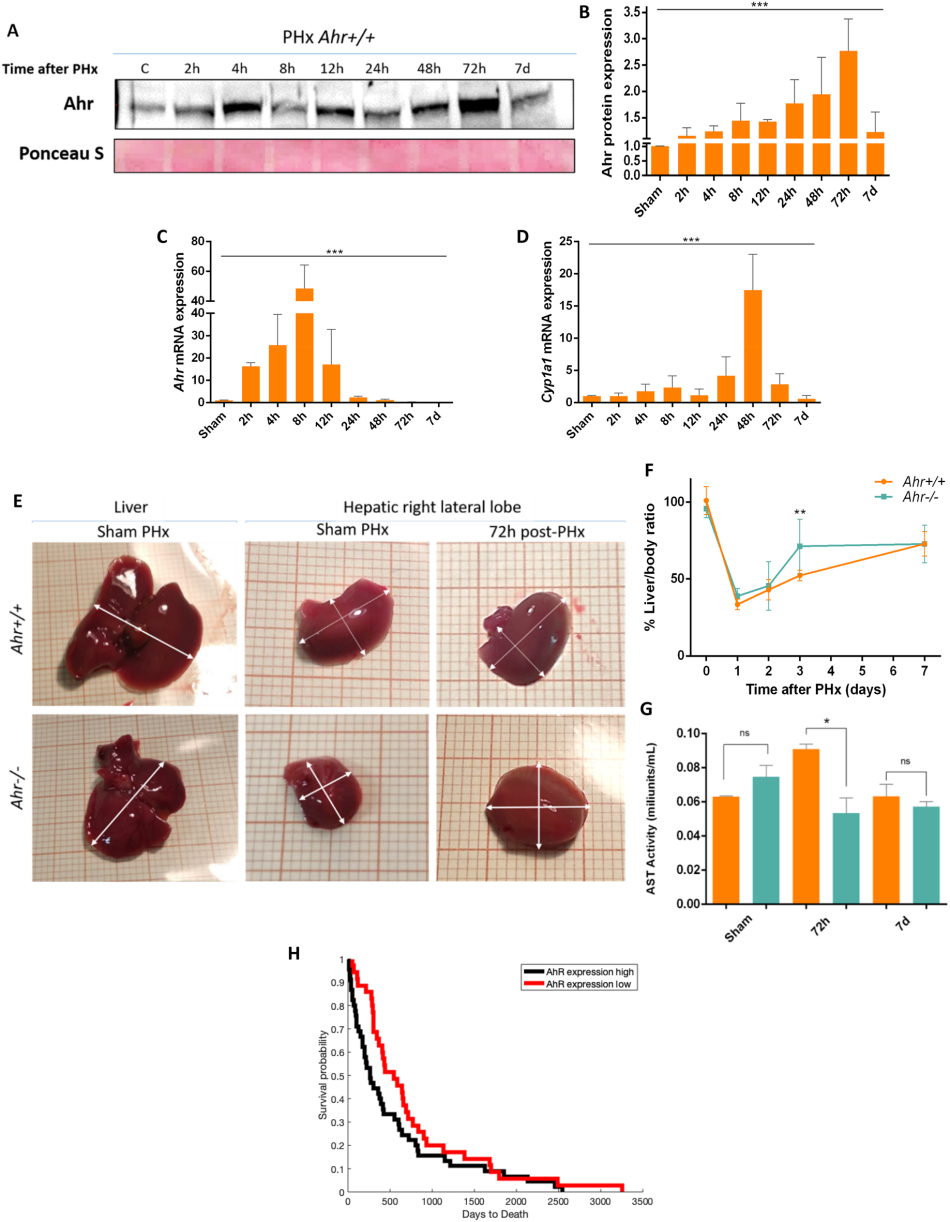
Ahr is induced and activated in the liver after PHx. (**A**, **B**) Protein expression of Ahr was analyzed in liver extracts of *AhR*^+*/*+^ mice at the indicated time points after PHx by immunoblotting. Ponceau staining was used to normalize protein levels. (**C**, **D**) Profiles of *Ahr* mRNA expression (**C**) and of its transcriptional targets *Cyp1a1* (**D**) and *Cyp1b1* in *AhR*^+*/*+^ mice at the indicated time points after PHx using RT-qPCR*. Gapdh* levels were used to normalize target gene expression (ΔCt) and 2^−ΔΔCt^ to calculate changes in mRNA levels with respect to sham-surgery wild type mice. Oligonucleotides and antibodies used are indicated in Supplementary Tables 1 and 3, respectively. (**E**) Lack of Ahr increases liver regeneration after PHx. Representative images of *Ahr*^+*/*+^ and *Ahr*^*−/−*^ livers before (left) and 72 h after 2/3 PHx (right); sham operated mice are shown by comparison (middle). (**F**) Regeneration was quantified as the percentage of liver *vs* total mouse weight. (**G**) AST activity were measured using serum samples from the same mice and under the same experimental conditions. (**H**) Kaplan–Meier survival of human patients with hepatocarcinoma after resection surgery according to Ahr expression. **p < 0.01, ***p < 0.001. Data are shown as mean ± SD. The experiments were performed using six (**E**, **F**) or three (**A**–**D**, **G**) mice for each genotype and three technical replicates were done.

To investigate the role of Ahr in hepatic regeneration, we first analyzed if 2/3 PHx (hereafter PHx) could alter its expression levels in *Ahr*^+*/*+^ mice. Protein and mRNA levels of Ahr gradually and transiently increased in the liver after 2/3 HPx (Fig. 1A–C, Fig. S1A,B). *Ahr* mRNA expression reached maximum values at 8 h (Fig. 1C) while protein levels steadily increased up to 3 days after PHx (Fig. 1A,B, Fig. S1A). mRNA and protein expression rapidly decreased to control pre-PHx values. To assess the functionality of the receptor, we analyzed the expression of its transcriptional targets *Cyp1a1* and *Cyp1b1*, whose levels also increased reaching maximum values at 24–48 h after PHx, to decrease to values like those pre-PHx (Fig. 1D,E). As expected, Ahr-null mice did not show any Ahr protein in the liver before or after PHx (Fig. S1B). These results indicated that Ahr transcription was induced by PHx in a transient manner and that the receptor protein subsequently produced was transcriptionally active following a physiological regenerative response in the absence of exogenous ligand.

### Ahr depletion accelerates liver regeneration after PHx

Macroscopic *de visu* examination of livers 72 h after PHx did not reveal significant differences between *Ahr*^+*/*+^ and *Ahr*^−/−^ mice or with respect to sham-operated mice (Fig. 1F). No differences were neither observed by microscopic analysis of liver sections obtained 48 h after PHx and stained for H&E (Supplementary Fig. S2). If any, an expansion of the Disse's space to favour the exchange between blood and hepatocytes could be appreciated in livers of both genotypes after PHx (Supplementary Fig. S2). Earlier reports have shown that *Ahr*^−/−^ adult mice have a significant reduction in liver weight^18,19^ (Fig. **1**F). Despite this basal phenotype, the kinetic of liver regeneration revealed that the liver to body weight ratio of *Ahr*^−/−^ mice was larger than that of *Ahr*^+/+^ mice particularly significant at 3 days after PHx, although both genotypes reached similar liver weight at the end of the regenerative process (Fig. 1F). A similar result was appreciated when measuring regeneration using the diagonal/lateral ratio of the right lateral lobe of the liver, which was higher in *Ahr*^−/−^ than in *Ahr*^+*/*+^ hepatectomized mice as compared to sham-operated controls for each genotype (Supplementary Fig. S3C). In addition, at 72 h and 7 days after PHx in which *Ahr*^−/−^ mice had recovered liver mass, we found that liver functionality had also been restored since AST levels were at baseline levels, while in *Ahr*^+*/*+^ mice at 72 h these levels still remained elevated (Fig. 1G). Giving a translational value to the study, we analyzed the correlation of Ahr expression with survival patients with surgeried hepatocarcinoma from TCGA by classifying these samples in Ahr-low or -high expression (see materials and methods). We observed that resected patients with Ahr high expression had a statistically significant worse survival (P = 0.02) as compared to patients with Ahr low expression (Fig. 1H).

**Figure 2:**
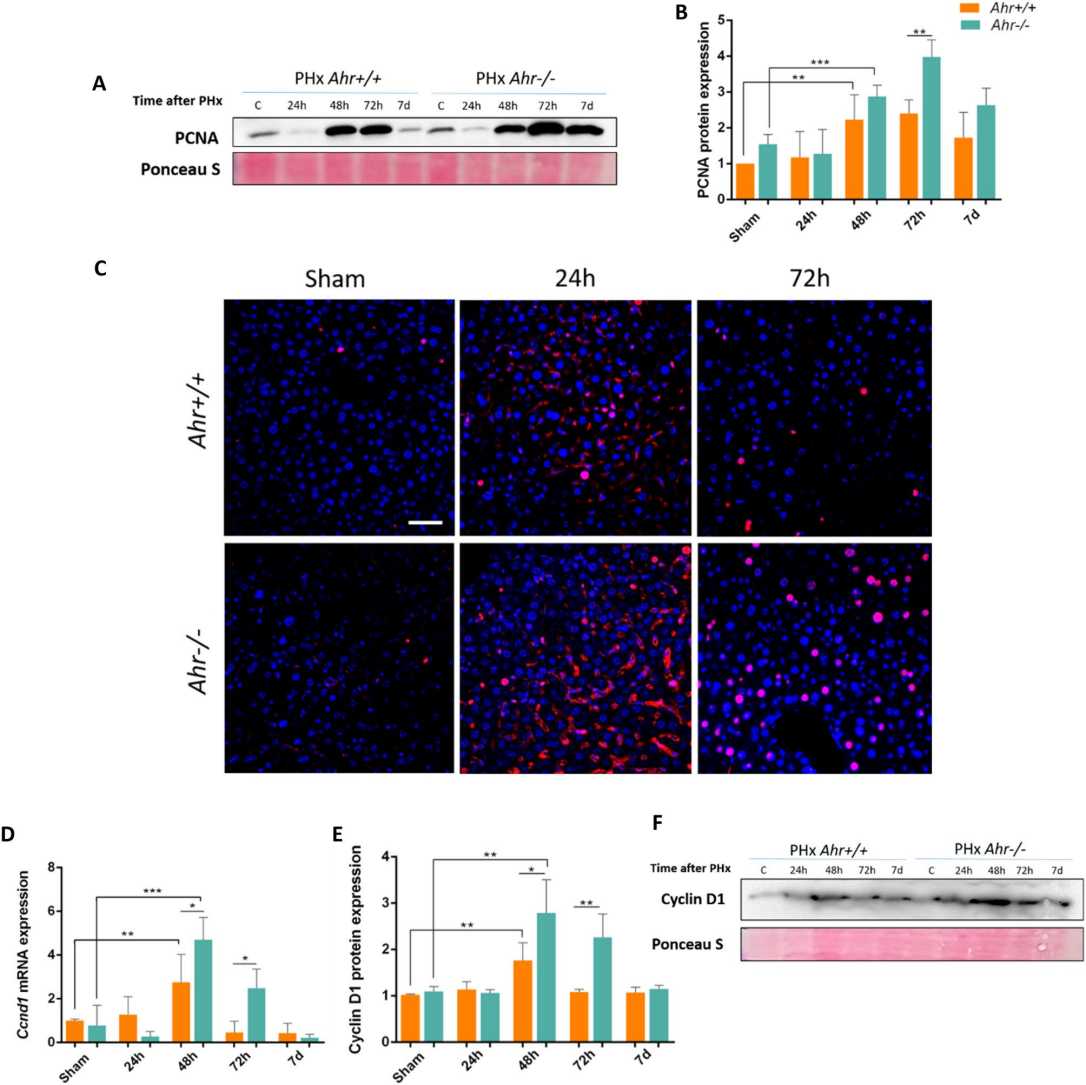
Proliferation rates are increased in the livers of *Ahr*^*−/−*^ mice after PHx. (**A**–**C**) Protein expression of PCNA was determined by immunoblotting (**A**, **B**) and immunofluorescence (**C**) in liver extracts of *Ahr*^+*/*+^ and *Ahr*^*−/−*^ mice at the indicated time points after PHx. Cyclin D1 expression was also analyzed at the mRNA level by quantifying *Ccnd1* by RT-qPCR (**D**) and at the protein level by immunoblotting (**E**, **F**) under the same experimental conditions. Ponceau staining was used to normalize protein levels. *Gapdh* was used to normalize target gene expression (ΔCt) and 2^−ΔΔCt^ to calculate changes in mRNA levels with respect to sham-surgery mice. Hoechst was used for staining cell nuclei. An Olympus FV1000 confocal microscope and the FV10 software (Olympus) were used for the analysis. Scale bar corresponds to 20 μm. *p < 0.05, **p < 0.01, ***p < 0.001. Data are shown as mean ± SD. The experiments were performed using three mice per time point and three technical replicates were done. Oligonucleotides and antibodies used are indicated in Supplementary Tables 1 and 3, respectively.

**Figure 3:**
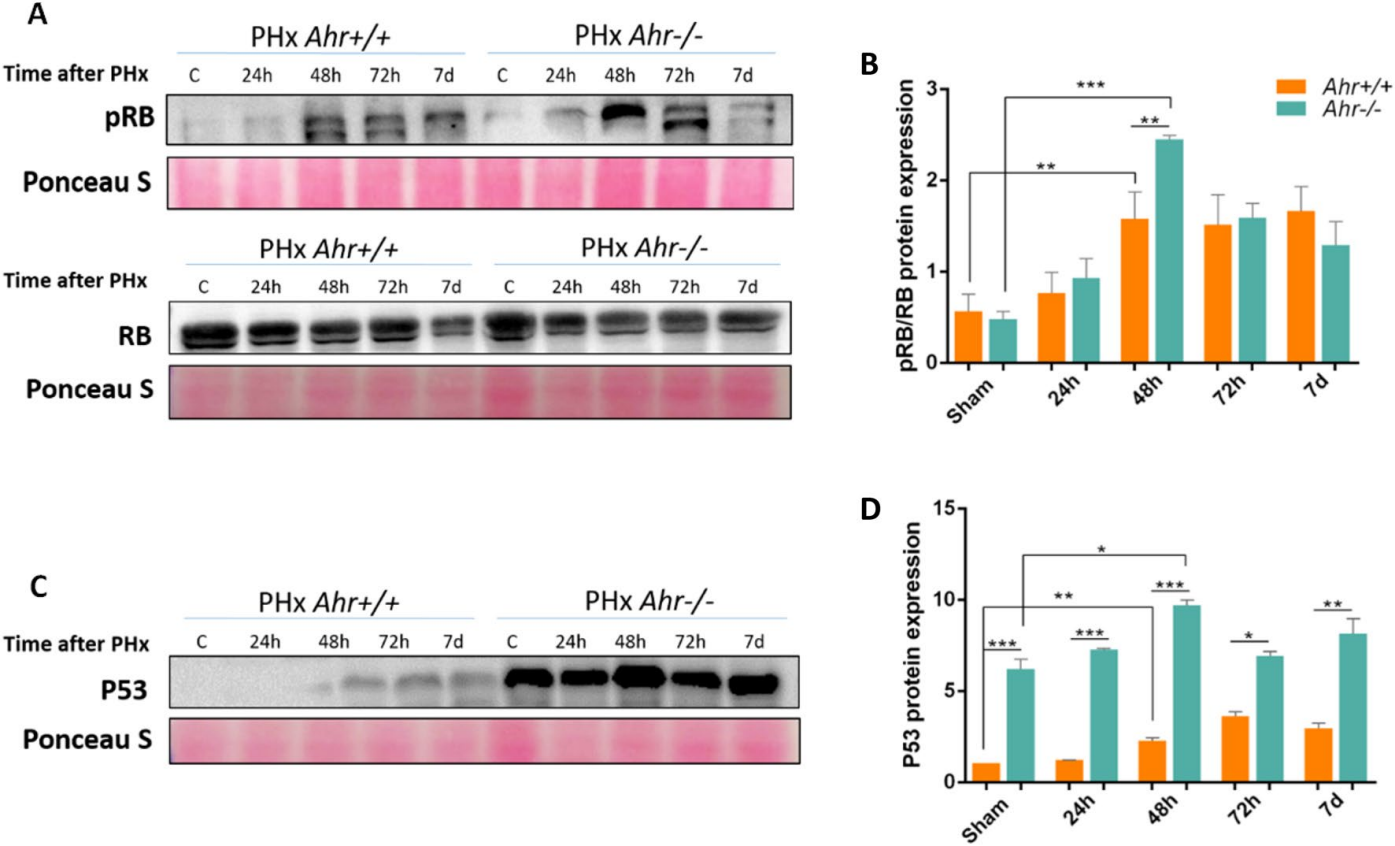
Proliferation rates are increased in livers of *Ahr*^*−/−*^ mice after PHx. Protein expression of pRb (Ser 807/811) and Rb as determined by immunoblotting (**A**) and quantification of the pRb/Rb ratio (**B**) in liver extracts of *Ahr*^+*/*+^ and *Ahr*^*−/−*^ mice at the indicated time points after PHx. (**C**, **D**) Protein expression and densitometric quantification of p53 under the same experimental conditions. Ponceau staining was used to normalize protein levels. *p < 0.05, **p < 0.01, ***p < 0.001. Data are shown as mean ± SD. The experiments were performed using three mice per time point and three technical replicates were done. Antibodies used are indicated in Supplementary Table 3.

Restoring liver tissue after PHx depends mostly on the proliferation of the remaining hepatocytes rather than on liver stem cells^4,6^. We next analyzed differences in the proliferative potential of *Ahr*^+*/*+^ and *Ahr*^−/−^ livers during PHx from 24 h to 7 days (Fig. 2). Results showed that the expression of the proliferation marker Proliferating Cell Nuclear Antigen (PCNA) increased in both genotypes with similar profiles, although a greatest increase at 72 h post-PHx was found in *Ahr-/-* livers as compared to *Ahr*^+*/*+^ livers (Fig. 2A,B, Fig. S3A), in agreement with the increase in liver to body weight ratio found at 72 h in *Ahr*^−/−^ mice. In fact, PCNA levels were already higher in *Ahr*^−/−^ than in *Ahr*^+*/*+^ livers under basal sham conditions (Fig. 2B). Immunofluorescence experiments further supported a larger number of cells with nuclear PCNA in *Ahr*^−/−^ than in *Ahr*^+*/*+^ livers at 72 h after PHx (Fig. 2C). At early stages after PHx (e.g. 24 h) livers of both genotypes had a certain amount of cytosolic PCNA protein (Fig. 2C) possibly resulting from the nuclear to cytoplasmic shuttling that takes place to protect from apoptosis and to sustain cell survival^23,24^. Furthermore, this could be an indicator of cells in G1 phase^25^. The additional proliferation marker Cyclin D1 was also overexpressed at the mRNA (Ccnd1, Fig. 2D) and protein levels (Fig. 2E,F, Fig. S3B) in *Ahr*^−/−^ livers with a pattern having a maximum at 48–72 h after PHx. Consistently, normalized phosphorylation levels (pRb/Rb) of the cell cycle regulatory protein Retinoblastoma were significantly higher in *Ahr*^−/−^ than in *Ahr*^+*/*+^ livers at 72 h after PHx (Fig. 3A,B, Fig. S4A). In addition, p53 protein, an important factor that positively modulates cell division after hepatectomy^26^ and that we found to be upregulated in undifferentiated adult AhR-null liver^21^, was significantly overexpressed in *Ahr*^−/−^ livers at all times after PHx, with a maximum level at 48 h (Fig. 3C,D, Fig. S4B).

### Lack of Ahr affects activation of the innate immune response in the priming phase of hepatic regeneration

**Figure 4:**
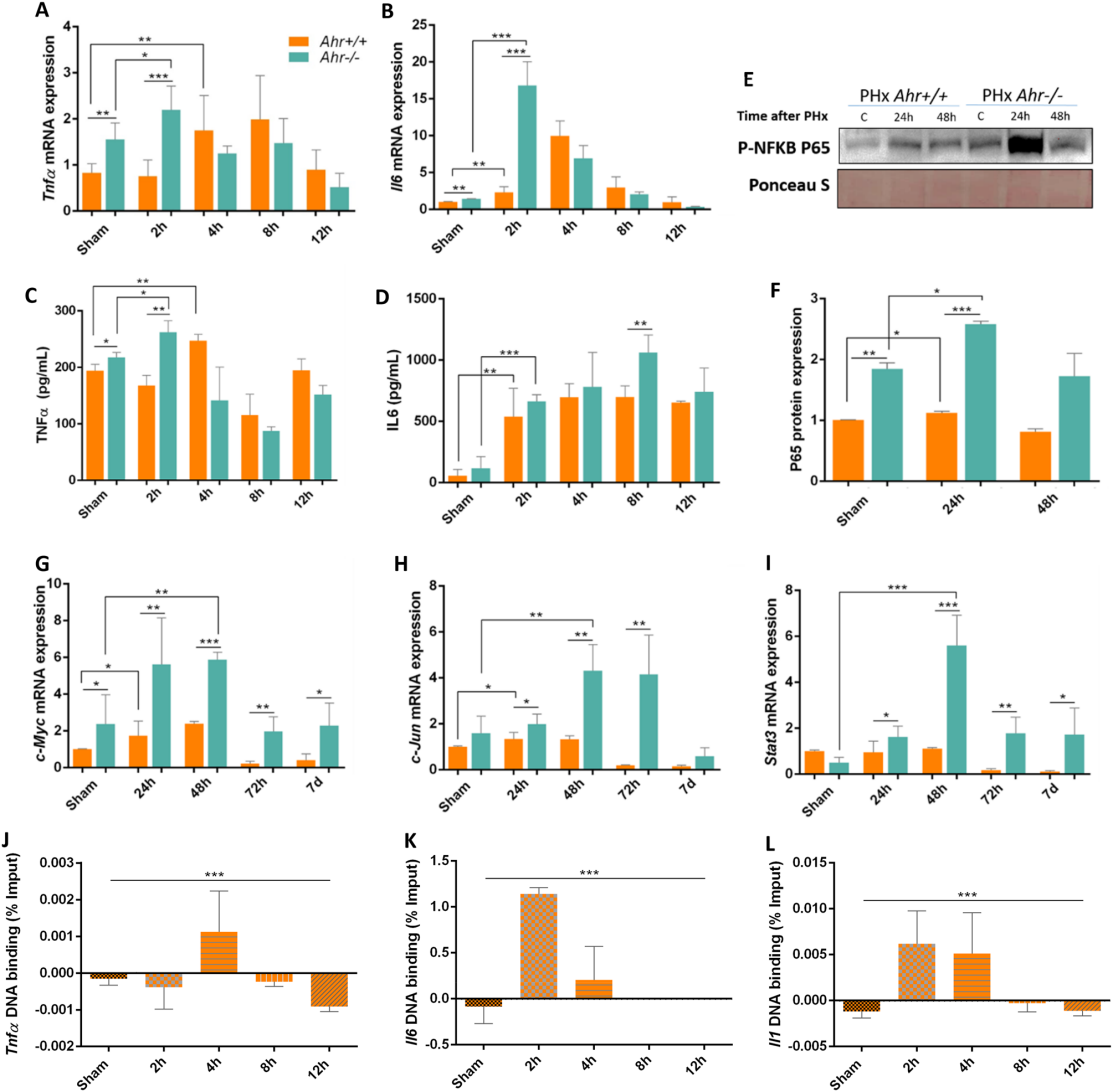
Ahr deficiency improves the cytokine network during the "priming phase” of regeneration. mRNA levels for TNFα (**A**) and IL6 (**B**) were determined by RT-qPCR in *Ahr*^+*/*+^ and *Ahr*^*−/−*^ liver at the indicated times after 2/3 PHx. Cytokine levels for TNFα (**C**) and IL6 (**D**) were measured in serum samples from the same mice and under the same experimental conditions. (**E**) Protein expression of p-p65-NFκβ (Ser536) was analyzed in liver extracts of mice at the indicated time points after PHx by immunoblotting and quantified by densitometry (**F**). Ponceau staining was used to normalize protein levels. (**G**–**I**) mRNA expression of *c-Myc* (**G**), *c-Jun* (**H**) and *Stat3* (**I**) was quantified using RT-qPCR. *Gapdh* was used to normalize target gene expression (ΔCt) and 2^−ΔΔCt^ to calculate changes in mRNA levels with respect to sham-surgery control mice. (**J**–**L**) Chromatin immunoprecipitation (ChIP) for Ahr binding to XRE binding sites located in the promoter of *Tnfα* (**J**), *Il6* (**K**) and *Il1* (**L**) was performed. qPCR was used to quantify changes in DNA binding and the results were normalized to the corresponding input samples. *p < 0.05, **p < 0.01, ***p < 0.001. Data are shown as mean ± SD. The experiments were performed using three mice per time point and three technical replicates were done. Oligonucleotides for gene expression analyses and for ChIP are indicated in Supplementary Tables 1 and 2, respectively. Antibodies used are indicated in Supplementary Table 3.

To study potential mechanisms through which Ahr could participate in liver regeneration after PHx, we next analyzed cytokines known to be involved in the inflammatory response taking place at the initial stages of liver regrowth^27,28^. *Tnfα* and *Il6* mRNA expression were transiently upregulated at early time points (e.g. 2 h) after PHx more markedly in *Ahr*^−/−^ than in *Ahr*^+*/*+^ livers (Fig. 4A,B). Measurement of the serum concentration of TNFα and IL6 revealed that lack of Ahr increased circulating levels of both cytokines immediately after PHx (e.g. 2–8 h) being the effect significantly larger in *Ahr*^−/−^ mice (Fig. 4C,D). TNFα causes the activation of NF-kB, which translocates to the nucleus and activates the transcription of acute phase proteins and proliferative genes. Consistently, the expression of phosphorylated NF-κB (p65-Ser^536^) was higher in *Ahr*^−/−^ than in *Ahr*^+*/*+^ livers with maximum protein levels at 24 h after PHx (Fig. 4E,F, Fig. S5). IL6 activates the JAK/STAT pathway and induces the expression of immediate early response oncogenes including *c-Myc, c-Jun* and *Stat3* which are responsible for the initiation of cell cycle^29^. We observed that *c-Myc*, *c-Jun* and *Stat3* were overexpressed at the mRNA level in *Ahr*^−/−^ livers after PHx with a maximum expression ranging from 24 to 48 h (Fig. 4G–I).

Since Ahr is a well-known transcription factor, we next analyzed if this receptor is needed to maintain transcriptional levels of *Tnfα*, *IL6* and *IL1* in wild type mice. Chromatin immunoprecipitation (ChIP) experiments revealed that Ahr binds to the promoter region of those three genes early after PHx (Fig. 4J–L), suggesting that this receptor serves to control physiological levels of these cytokines in response to PHx, and that lack of Ahr may produce an increase in their expression and activity. Therefore, Ahr deficiency likely improves the cytokine network during the "priming phase” of liver regeneration.

### Pluripotency markers are modulated in an Ahr-dependent manner during liver regeneration

**Figure 5:**
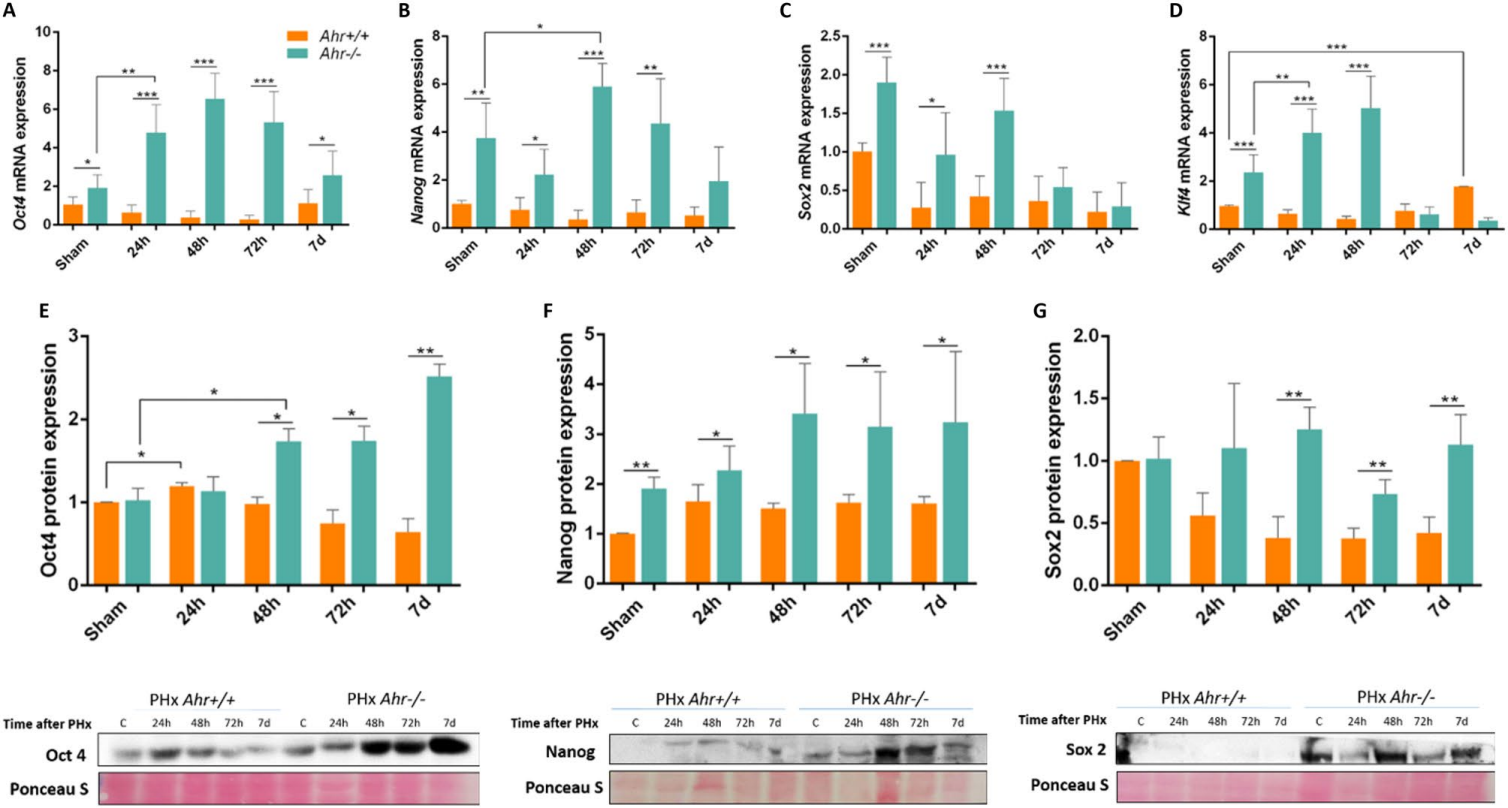
Pluripotency markers are modulated in an Ahr-dependent manner during liver regeneration. (**A**–**D**) mRNA expression of *Oct4* (**A**), *Nanog* (**B**), *Sox2* (**C**) and *Klf4* (**D**) was determined in *Ahr*^+*/*+^ and *Ahr*^*−/−*^ livers at the indicated times after PHx by RT-qPCR. (**E**–**G**) Protein expression of Oct4 (**E**), Nanog (**F**) and Sox2 (**G**) was analyzed in liver extracts of *Ahr*^+*/*+^ and *Ahr*^*−/−*^ mice at the indicated points after PHx by immunoblotting. Ponceau Staining was used to normalize protein levels. *Gapdh* was used to normalize target gene expression (ΔCt) and 2^−ΔΔCt^ to calculate changes in mRNA levels with respect to sham-surgery control mice. *p < 0.05, **p < 0.01, ***p < 0.001. Data are shown as mean ± SD. The experiments were performed using three mice per time point and three technical replicates were done. Oligonucleotides and antibodies used are indicated in Supplementary Tables 1 and 3, respectively.

Ahr has been identified as a regulator of pluripotency markers OCT4, NANOG^21,30^ and SOX2^31^. We next analyzed the expression of these factors in *Ahr*^+*/*+^ and *Ahr*^−/−^ livers subjected to PHx. mRNA expression experiments revealed that *Oct4*, *Nanog, Sox2* and *Klf4* significantly increased their expression in *Ahr*^−/−^ mice during liver regeneration as compared to their levels *Ahr*^+*/*+^ mice (Fig. 5A–D). Protein expression analyses also showed increased amounts of Oct4, Nanog and Sox2 in *Ahr*^−/−^ livers (Fig. 5E–G, Fig. S6A–C). Consistently, the pattern of protein expression for these markers trailed their mRNA profiles after PHx, particularly in *Ahr*^−/−^ livers. In addition, whereas mRNA levels of *Oct4, Nanog* and Sox2 markedly decayed at the end of regeneration in absence of Ahr, their protein expression remained significantly higher even at 7 days post-PHx (Fig. 5). Thus, Ahr deficiency induces an undifferentiated and pluripotent status in the liver that increases after PHx and that could improve its regenerative potential.

### Lack of Ahr expands the side population in the regenerating liver

**Figure 6:**
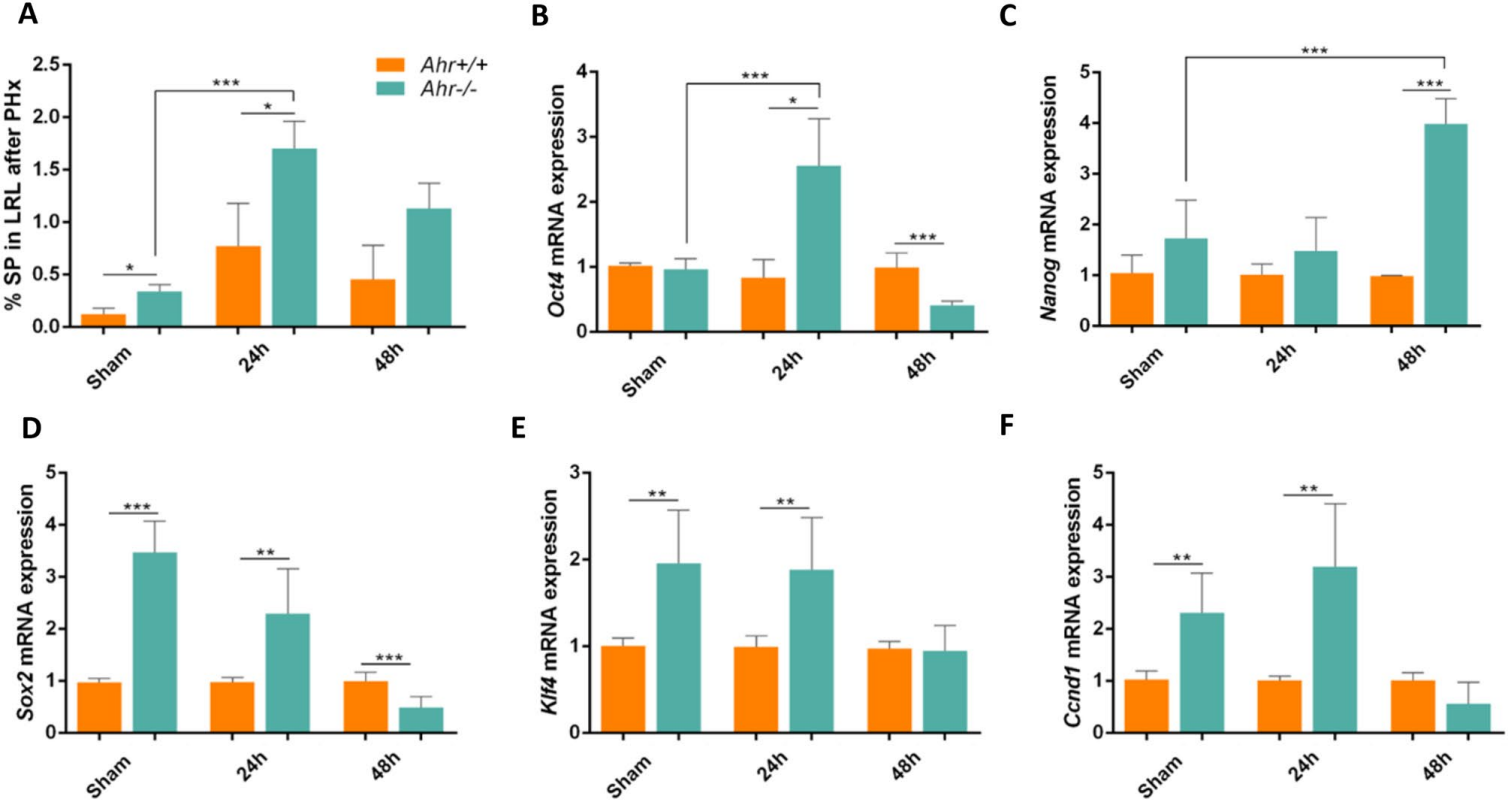
Lack of Ahr expands the side population and upregulates pluripotency markers in the regenerating liver. (**A**) Lateral right lobes of the liver were obtained from of *Ahr*^+*/*+^ and *Ahr*^*−/−*^ mice at the indicated points after PHx. Samples were processed to isolate and quantify side population cells (SP) by cell sorting. SP + cells are expressed with respect to the total number of liver cells. (**B**–**F**) mRNA was purified from SP + cells of both genotypes and used to analyze by RT-qPCR the expression of *Oct4* (**B**), *Nanog* (**C**), *Sox2* (**D**), *Klf4* (**E**) and *Ccnd1* (**F**). *Gapdh* was used to normalize target gene expression (ΔCt) and 2^−ΔΔCt^ to calculate changes in mRNA levels with respect to wild-type mice. *p < 0.05, **p < 0.01, ***p < 0.001. Data are shown as mean ± SD. The experiments were performed using three mice per time point and three technical replicates were done. Oligonucleotides used are indicated in Supplementary Table 1.

In addition to the hepatocytes, liver stem cells also proliferate and expand to help restore hepatic mass and functionality^29^. We thus determined the side population cells present in *Ahr*^+*/*+^ and *Ahr*^−/−^ livers as an indication of their content in undifferentiated cells with stem-like phenotype. We found a transient expansion of side population cells and at early time points after PHx in both genotypes, being the increase significantly higher in *Ahr*^−/−^ livers (Fig. 6A, Fig. S7A). Furthermore, the number of total liver cells which expressed undifferentiation CD133 + EpCam + markers was higher in *Ahr*^−/−^ mice (Fig. S7B). As the stem-like phenotype has been linked to pluripotency factors *Oct4*, *Nanog*, *Sox2* and *Klf4*, we analyzed the mRNA expression of these factors in side population cells. We observed a significant increase in the mRNA levels of *Oct4* (Fig. 6B) and Nanog (Fig. 6C) at 24 h at 48 h after PHx in SP cells isolated from *Ahr*^−/−^ livers, respectively. *Sox2 and Klf4* mRNAs were already at higher levels under basal conditions (sham-operated mice) in *Ahr*^−/−^ SP cells to gradually decrease to control values at 48 h after PHx (Fig. 6D,E). Additionally, the cell cycle positive regulator *Ccnd1* gene was also upregulated under basal conditions and at the initial stages after PHx in *Ahr*^−/−^ SP cells (Fig. 6F). Thus, a higher percentage of cells with a stem-like phenotype in *Ahr*^−/−^ livers under basal conditions likely improves the regenerative potential of these mice.

### Ahr-null livers show upregulation of Hippo and Wnt signalling pathways

**Figure 7:**
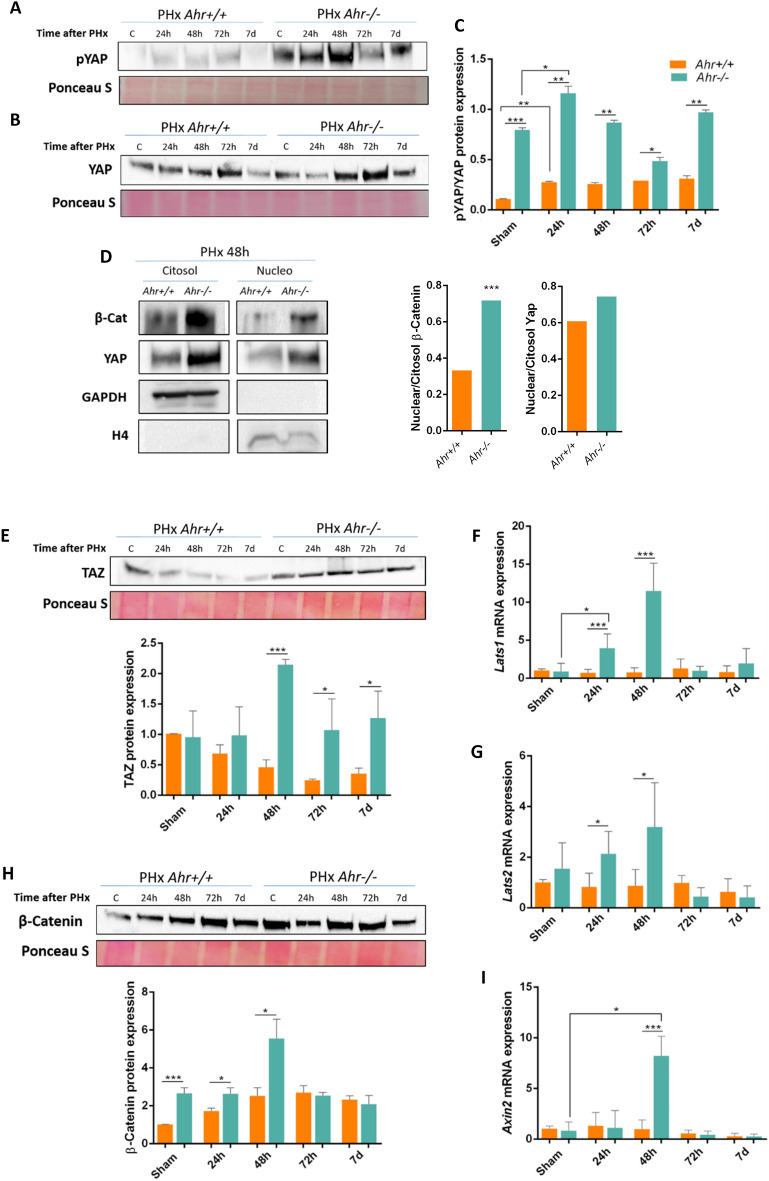
Ahr-null livers show deregulation of Hippo and Wnt signalling pathways. p-YAP protein expression (**A**) and p-YAP (**B**) was analyzed in liver extracts of *Ahr*^+*/*+^ and *Ahr*^*−/−*^ liver samples at the indicated points after PHx by immunoblotting. Cytosolic and nuclear levels of β-Catenin and YAP were determined by immunoblotting using specific antibodies (**D**). GAPDH and Histone H4 were used as controls for cytosolic and nuclear protein, respectively. Protein expression of the Hippo pathway effector TAZ (**E**) and mRNA expression of the YAP and TAZ kinases *Lats1* (**F**) and *Lats2* (**G**). Protein levels of β-Catenin (**H**) were analyzed by immunoblotting whereas the β-Catenin pathway transcriptional target *Axin2* **(I**) was quantified using RT-qPCR under the same experimental conditions. *Gapdh* was used to normalize target gene expression (ΔCt) and 2^−ΔΔCt^ to calculate changes in mRNA levels with respect to sham-surgery control mice. Ponceau staining was used to normalize protein levels. *p < 0.05, **p < 0.01, ***p < 0.001. Data are shown as mean ± SD. The experiments were performed using three mice per time point and three technical replicates were done. Oligonucleotides and antibodies used are indicated in Supplementary Tables 1 and 3, respectively.

The Hippo pathway plays a fundamental role in controlling organ size, maintaining the stem cell population and in regulating cell differentiation^32,33^. Furthermore, the fate between hepatic progenitors and hepatocytes is also controlled by the Hippo pathway. Protein expression of the Hippo transcriptional factor Yap increased upon PHx in the liver of mice of both genotypes (Fig. 7A, Fig. S8A), although the levels of phosphorylated Yap (p-Yap) were higher under basal conditions and in response to PHx in *Ahr*^−/−^ than in *Ahr*^+*/*+^ livers (Fig. 7B, Fig. S8A). Consequently, the p-Yap/Yap ratio remained significantly larger in Ahr-null mice than in wild type animals before (sham-operated) and during regeneration following PHx (Fig. 7C, Fig. S8B). Yap phosphorylation induces its retention in the cytosol, but the ratio of nuclear vs cytosolic Yap was similar in both genotypes (Fig. 7D, Fig. S9B). The additional Hippo pathway transcriptional effector Taz showed a significant increase in protein levels from 48 h to 7 days in *Ahr*^−/−^ livers (Fig. 7E, Fig. S9A). Interestingly, protein kinases responsible for Yap phosphorylation *Lats1* and *Lats2* were markedly upregulated at the mRNA level in the absence of Ahr at times after PHx consistent with the increase in p-Yap/Yap observed (Fig. 7F,G).

β-Catenin expression increased after PHx in livers of both genotypes although the levels reached early after PHx (e.g. 48 h) were significantly higher in *Ahr*^−/−^ than in *Ahr*^+*/*+^ livers (Fig. 7H, Fig. S8A). Intracellular distribution of β-Catenin revealed that its nuclear amounts were increased in Ahr-null with respect to Ahr-expressing livers (Fig. 7D, Fig. S8B), suggesting an increased transcriptional activity. Consistently, the mRNA expression of the β-Catenin transcriptional target *Axin2* largely increased in *Ahr*^−/−^ livers at the same times after PHx at which β-Catenin protein expression and nuclear localization were enhanced (Fig. 7I, Fig. S8B). These results suggest that Ahr deficiency upregulates the Hippo and Wnt/β-Catenin pathways, probably contributing to their improved response to PHx.

### Different changes in metabolic profile during liver regeneration in absence of Ahr

**Figure 8:**
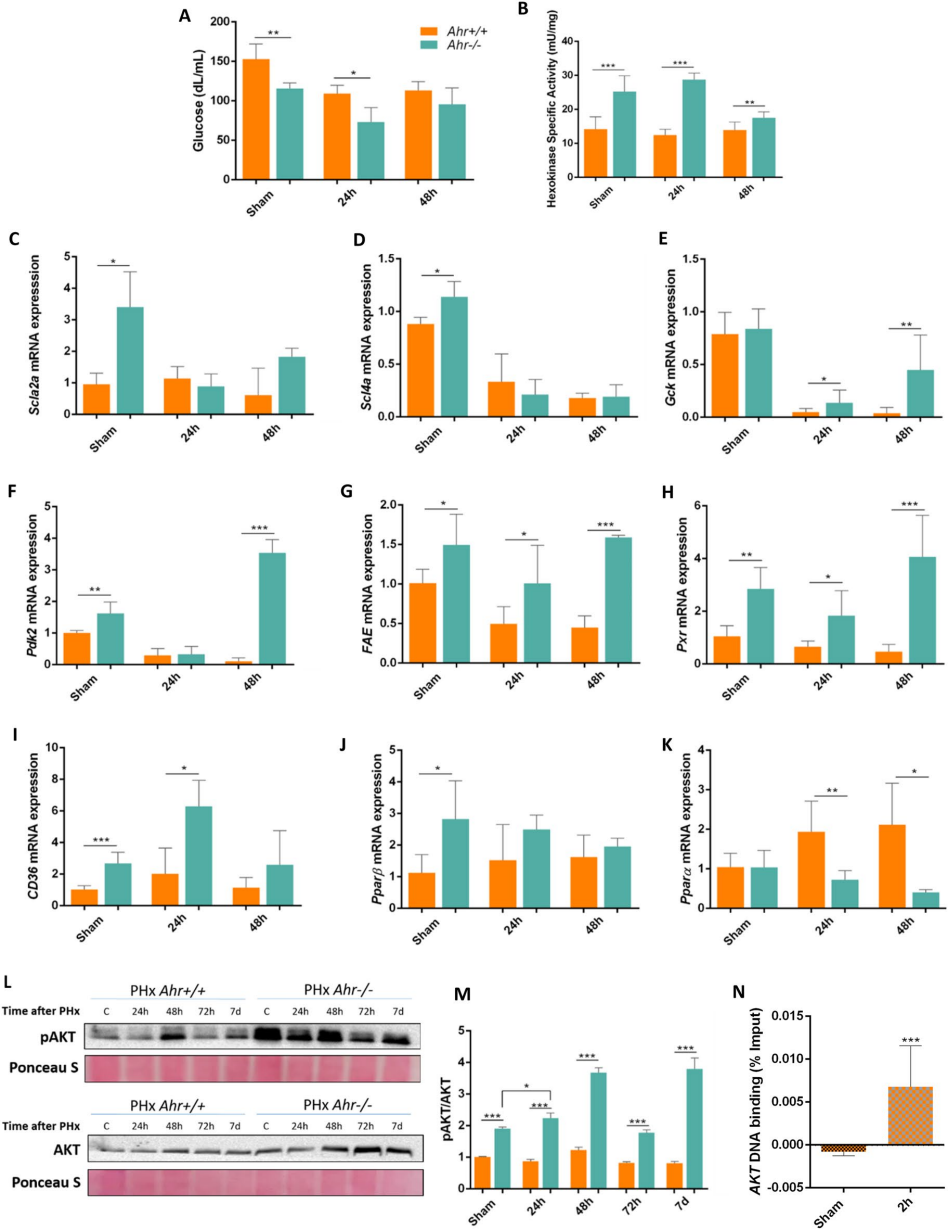
Metabolic profile during liver regeneration in absence of Ahr. (**A**) Blood glucose concentration in *Ahr*^+*/*+^ and *Ahr*^*−/−*^ mice at the indicated points after PHx was measured using a glucometer. (**B**) Hexokinase activity in liver homogenates under the same experimental conditions. mRNA expression for *Slc2a2* (**C**), *Scl2a4* (**D**), *Gck* (**E**), *Pdk2* (**F**), *Fae* (**G**), Pxr (**H**), *Cd36* (**I**), *Pparb* (**J**) and *Pparα* (**K**) was analyzed by RT-qPCR in liver extracts of *Ahr*^+*/*+^ and *Ahr*^*−/−*^ mice after PHx. Protein expression of Akt and p-Akt (**L**) was analyzed by immunoblotting and the p-Akt/Akt ratio quantified (**M**). Ahr binding to the XRE binding sites located in the *Akt* gene promoter was determined by chromatin immunoprecipitation (ChIP) under the same experimental conditions (**N**). qPCR was used to quantify changes in DNA binding and the results were normalized to the corresponding input DNAs. *Gapdh* was used to normalize target gene expression (ΔCt) and 2^−ΔΔCt^ to calculate changes in mRNA levels with respect to sham-surgery control mice. *p < 0.05, **p < 0.01, ***p < 0.001. Data are shown as mean ± SD. The experiments were performed using three mice per time point and three technical replicates were done. Ponceau staining was used to normalize protein levels.

Partial hepatectomy involves a significant loss of glycogen, which is essential for hepatic metabolism^34^. During the initial phases of liver regeneration, the amount of glucose in the blood is reduced and an alternative source of energy such as lipids is necessary^35^. As expected, the amount of glucose in the blood was reduced in mice of both genotypes after PHx. However, *Ahr*^−/−^ mice had significantly lower levels of glucose already under basal conditions (sham-operated) and after 24 h of PHx than *Ahr*^+*/*+^ mice (Fig. 8A). The enzymatic activity of hexokinase, which is mainly responsible for glucose phosphorylation in non-parenchymal cells^36^, including liver progenitor cells^37^, was significantly increased in *Ahr*^−/−^ mice before and up to 48 h after PHx (Fig. 8B). These differences could be due to a more efficient basal glucose transport in *Ahr*^−/−^ livers since the expression of the main glucose transporters *Slc2a2* and *Scl2a4* were overexpressed as compared to *Ahr*^+*/*+^ livers (Fig. 8C,D). Additionally, the expression of genes involved in glucose metabolism such as *Gck* and *Pdk2* was reduced at 24 h after PHx to then recover at 48 h with a more pronounced pattern in *Ahr*^−/−^ than in *Ahr*^+*/*+^ livers (Fig. 8E,F).

The differences in glycolytic metabolism between both genotypes could be related to an altered lipid metabolism. This led us to analyze the expression of genes responsible for regulating lipogenesis, β-oxidation and lipid transport. mRNA expression of the long-chain free fatty acid elongase, *Fae*, the Pregnane-X Receptor, *Pxr*, and the free fatty acid transporter, *Cd36* was increased in *Ahr*^−/−^ livers under basal conditions (sham-operated) (Fig. 8G–I). Upon PHx, *Fae* and *Pxr* exhibited a transient downregulation that returned to basal values at 48 h (Fig. 8G,H). *Cd36*, on the contrary, was upregulated at shorter times (e.g. 24 h) to decrease to initial levels at 48 h (Fig. 8I). Additional regulators of lipid metabolism such as *Pparα* and *Pparβ*^38^ were also analyzed. Whereas *Pparβ* showed no significant differences in expression between genotypes following PHx (Fig. 8J), *Pparα* was upregulated in *Ahr*^+*/*+^ livers after PHx as compared to *Ahr*^−/−^ livers (Fig. 8K), thus producing an expression profile opposed to that of *Fae*, *Pxr*, *CD36* and *PParβ*. AKT is an additional factor involved in signalling pathways controlling hepatic metabolism through mTORC1^39,40^. Immunoblotting analysis revealed an increase in the pAKT/AKT ratio in *Ahr*^−/−^ livers under basal conditions and up to 7 days following PHx (Fig. 8L,M, Fig. S9B). Moreover, by using chromatin immunoprecipitation we found that Ahr binds to the *Akt* gene promoter in *Ahr*^+/+^ liver very early (e.g. 2 h) after PHx (Fig. 8N). Collectively, these data suggest that Ahr deficiency increases the glycolytic and lipid metabolism in response to partial hepatectomy and that such response likely increases their regeneration capability in the liver.

## Discussion

The liver is the main organ responsible for detoxification and, as a result, it is constantly exposed to damaging events produced by a wide variety of toxic compounds. Remarkably, the liver can react to such injuries by activating a regenerative process intended to restore tissue architecture and function. In fact, a complete liver regeneration can be achieved after exposure to necrosis-inducing agents or following partial hepatectomy in mammals^4,5^. From a human perspective, knowing the mechanisms driving liver regeneration is essential to improve survival of patients undergoing liver resection. We have shown that Ahr is required to adjust liver regeneration after acute toxic injury^22^ and we and others have reported that Ahr serves to reduce hepatocarcinogenesis burden caused by postnatal exposure to diethylnetrosamine in mice^22,41^. Moreover, previous work also suggest that the higher regenerative potential and increased susceptibility to carcinogenesis of Ahr-null mice could be due to their more undifferentiated and pluripotent phenotype in different organs including liver^21,22,30,42–45^. In this study, we have investigated potential mechanisms through which Ahr adjusts liver regeneration in response to a massive tissue removal resembling that used in humans. Our main claim is that Ahr is required during the early “priming phase” of liver regeneration to control the expansion of proliferative cells with pluripotent characteristics and the activation of signalling pathways modulating cell differentiation and metabolism. Consequently, Ahr seems to regulate re-entry of hepatocytes from their quiescent state into proliferative cell cycle.

Independent studies have shown that Ahr participates in the G1/S phase of the cell cycle in hepatocytes. The Ahr ligand TCDD impairs liver regeneration after PHx by controlling the levels of p21^Cip1^ and p27^Kip1^^46^. In addition, sustained Ahr activation causes cell cycle arrest in murine Hepa-1c1c7 hepatoma cells^47,48^. Thus, Ahr activity may be adjusted in the liver to control proliferation and regeneration eventually avoiding organ overgrowth. Interestingly, the expression and transcriptional activity of Ahr markedly increased during and at the onset of regeneration, suggesting its role in limiting liver growth and supporting a more unconstrained regenerative process in *Ahr*^−/−^ mice. Consistently, the expression of cell cycle regulators Cyclin D1 and PCNA rapidly increased in *Ahr*^−/−^ livers. Absence of Ahr may also favour release of E2F proteins from inactivated pRb and cell cycle entry given that Ahr can form complexes with pRb to stabilize its active conformation and to block cell cycle progression^49,50^.

p53 regulates p21^Cip1^ transcription^51^ as well as the transcription of genes involved in glycolysis, lipogenesis and other metabolic pathways^52^. p53 is also involved in preventing polyploidy of mature cells^26,53^ and, notably, Ahr deficiency sustains an undifferentiated diploid phenotype in the adult liver with upregulation of p53^21^. Since diploidy is associated to a proliferative (undifferentiated) status in the liver, the higher levels of p53 found in *Ahr*^−/−^ mice upon PHx may contribute to their enhanced regenerative potential.

Acute inflammation induced after liver surgery also triggers cell cycle entry^28^. Kupffer cells secrete TNF-α and IL6 during the “priming phase” of regeneration^54^, which induce multiple genes coding for acute phase proteins that facilitate the proliferation of hepatocytes^5^. Ahr has known roles in the immune system including the limitation of macrophage´s responses to inflammatory stimuli upon Ahr activation^55^. *Ahr*^−/−^ mice had higher inflammatory levels after PHx coincident with enhanced TNF-α and IL6 released into the serum. Chromatin immunoprecipitation revealed that Ahr transcriptionally regulates *Tnfα*, *Il6* and *Il1* genes during HPx-induced regeneration. Furthermore, Ahr-null mice also had upregulation of NF-κB and enhanced transcription of its target genes involved in the proliferation of hepatocytes such as *c-Myc*, *c-Jun* and *Stat3*.

Accelerated regeneration in *Ahr*^−/−^ mice could be due to the activation and expansion of undifferentiated stem-like cells residing in the liver. A higher content of cells expressing stemness markers Oct4, Nanog, Klf4 and Sox2 were present under basal (pre-PHX) conditions and during regeneration in Ahr-null livers. Interestingly, these undifferentiated cells exhibited their highest expansion at the early stages of regeneration, suggesting that Ahr is needed to control stemness at the initial “priming phases” of liver recovery. This hypothesis is further supported by previous studies indicating that Ahr is a determining factor in controlling cell undifferentiation in diverse repairing processes including those regenerating the liver^22^ and the lung^56^ and even during NSCLC induced by the KRas-G12D human oncogene in an Ahr deficient background^44^.

The Hippo pathway is an additional signalling network relevant in liver regeneration. A functional interaction between Hippo and Ahr in the liver appears likely since Hippo transcriptional intermediate Yap becomes induced, phosphorylated and localized to the cytosol at a greater extent in hepatectomized *Ahr*^−/−^ than in *Ahr*^+/+^ mice, suggesting that Ahr controls the level of Hippo activation upon PHx-induced regeneration. Consistently, kinases for the cytosolic retention of Yap and Taz Lats1 and Lats 2 were also upregulated in the absence of Ahr. We suggest that Ahr contributes to maintain the correct Hippo pathway activation during liver regeneration.

Wnt/β-catenin signalling regulates proliferation of normal and tumour liver cells due to the control of pluripotency markers^14^. Wnt/β-catenin and Ahr pathways are coregulated in different physiological conditions, whereas Ahr represses Wnt/β-catenin in liver progenitor cells^57^, Wnt/β-catenin increases the expression of Ahr target genes in human hepatocytes^58^. Following PHx, levels of nuclear β-catenin were higher in *Ahr*^−/−^ than in *Ahr*^+/+^ livers, suggesting that such signalling was also contributing to enhanced proliferation and regeneration in the absence of receptor. This functional interaction is not restricted to mammals since Ahr activation also modulates Wnt/β-catenin signalling during fin regeneration in zebrafish^59^.

Liver regeneration involves a process of metabolic adaptation: in the early stages energy is obtained from lipolysis while in later stages glycolysis is the main source of energy^60^. Glucose concentration in the blood decreased to a greater extent in *Ahr*^−/−^ mice, which could be due to their higher expression of the liver-specific glucose transporter *Glut2*. Furthermore, the glycolytic metabolism was also increased in Ahr-null livers likely as a result of their upregulated hexokinase activity and increased expression of *Pdk2*. Furthermore, *Pdk2* and *Gck* expression recovered earlier in *Ahr*^−/−^ than in *Ahr*^+/+^ livers. In this regard, the improved AKT expression and phosphorylation found in Ahr deficient mice could underlay the existence of a process to restore glucose-dependent hepatic metabolism during regeneration. Maintenance of Hexokinase activity in *Ahr*^−/−^ livers short after PHx could be due to the expansion of stem cells with a preferential glycolytic metabolism^61^. A previous report has described the presence of hepatic steatosis in postnatal *Ahr*^−/−^ mice^19^. Genes involved in lipid metabolism such as *Pxr*, *Fae*, *Cd36* and *Pparβ* showed higher expression levels before and PHx in *Ahr*^−/−^ mice whereas *Pparα* had an opposite pattern increasing in *Ahr*^+/+^ livers*.* Since PPARα is relevant in the control of lipid metabolism^62^, its reduced expression in *Ahr*^−/−^ livers could help explain their lower lipidic metabolism.

In summary, physiological Ahr activity could be required for the proper initiation and termination of the proliferative responses driving liver regeneration. Ahr has an important role in regulating the induction of essential pluripotency and stemness factors and in adjusting Wnt/β-catenin and Hippo signalling pathways during the regenerative process triggered by a massive loss of liver tissue. A main finding is that lack of Ahr markedly upregulates the initial “priming phase” of liver regeneration, resulting in a more efficient process than that taking place under Ahr expression. This conclusion is further supported by the similar regenerative profile found after inducing acute toxic damage in the liver and lung^22,56^. Future work will be required to determine if pharmacological modulation of Ahr represents a promising strategy to accelerate liver regeneration in patients with different forms of liver damage including cancer-dependent partial hepatectomy.

## Materials and methods

### Mice and treatments

*Ahr*^+*/*+^ and *Ahr*^*−/−*^ female and male mice of the C57BL6/N x 129 Sv mixed genetic background^18^ at 10 to 14 weeks of age were subjected to 2/3 PHx by removing the left and median lobes of the liver essentially as described^63^. Surgical procedures were performed under 2% isoflurane anesthesia. Mice were sacrificed at 2 h, 4 h, 8 h, 12 h, 24 h, 48 h, 72 h, and 7 days after surgery and livers collected and processed for analysis. Control mice were subjected to sham surgery without removal of liver tissue. All work involving mice was performed in accordance with the National and European legislation (Spanish Royal Decree RD53/2013 and EU Directive 86/609/CEE as modified by 2003/65/CE, respectively) for the protection of animals used for research. Experimental protocols using mice were approved by the Bioethics Committee for Animal Experimentation of the University of Extremadura (Registry 109/2014) and by the Junta de Extremadura (EXP-20160506–1). This study is reported in accordance with ARRIVE guidelines. Mice had free access to water and rodent chow.

### Hematoxylin/eosin staining of liver sections

Liver tissues isolated at the indicated times after HPx were fixed overnight at room temperature in buffered formalin and included in paraffin. Sections of 3 μm were prepared, deparaffinized in xylol and gradually re-hydrated to phosphate buffered saline (PBS). Sections were incubated for 3 min with hematoxylin, washed with tap water and stained with eosin for 1 min. After a final washing step, sections were de-hydrated, mounted and observed in a NIKON TE2000U microscope equipped with 4× (0.10 numeric aperture), 10× (0.25 numeric aperture) and 20× (0.40 numeric aperture) objectives.

### Liver function test

Blood samples were collected in heparinized tubes and allowed to clot at 4 °C. The serum level of Aspartate Aminotransferase (AST) was measured using an Activity Assay kit according to the manufacturer’s instructions (Sigma-Aldrich).

### ELISA assays

Blood samples were collected in heparinized tubes and allowed to clot at 4 °C. Serum levels of IL6 and TNFα were measured using specific ELISA kits according to the manufacturer’s instructions (RayBiotech and Cloud-Clone, respectively).

### Side population (SP) isolation by cell sorting and cell undifferentiation staining

Side population (SP) stem-like cells were isolated from *Ahr*^+*/*+^ and *Ahr*^*−/−*^ livers essentially as described with some modifications^22^. Briefly, liver tissues were finely minced in PBS and rotated for 30 min at 37 °C in digestion solution (PBS containing 0.5 U/ml dispase and 60 U/ml collagenase, Invitrogen). Once digested, tissues were homogenized by passing through a 21-gauge syringe, filtered by a 0.40 μm mesh and centrifuged at 300 × g for 5 min. Pellets were then resuspended in sorting medium (PBS containing 10% FBS). To isolate SP liver cells, cellular pools were stained for 90 min at 37 °C with a solution containing 5 μg/ml Hoechst 33,342 (Sigma Aldrich). Cells were centrifuged and resuspended in HEPES-HBSS buffer and incubated with 50 μM Fumitremorgin C (Sigma Aldrich) to inhibit the ABCG2 extrusion pump. Propidium iodide (10 nM) was used to discriminate dead cells from the purification step. A MoFlo Astrium EQ flow cytometer (Beckman Coulter) was used.

Also, liver cells were stained for the undifferentiation markers CD133 PE and EpCam APC (Milteny Biotech) to quantify more undifferentiated stem cells. Briefly, 5 µl of each antibody were added to 1 million cells and incubated for 30 min at RT, after washing in PBS 0,1% BSA, cells were resupend in 500 µl PBS 0.1% BSA and analyzed in a Cytoflex S flow cytometer (BeckmanCoulter).

### Immunofluorescence

Liver sections (3 μm) were processed as indicated above without the blocking step. Unspecific epitopes were inactivated by 1 h incubation at room temperature in TBS-T containing 0.2% gelatin and 3% BSA. Sections were then incubated overnight at 4 °C with the corresponding primary antibodies diluted in PBS-T containing 0.2% gelatin, washed in the same gelatin solution and incubated for 1 h at room temperature with Alexa-488 or Alexa-633-labelled secondary antibodies. After washing the excess of secondary antibodies, sections were dehydrated, mounted on Mowiol and visualized using an Olympus FV1000 confocal microscope (Olympus). Objectives used were 10× (0.40 numeric aperture) and 20× (0.70 numeric aperture). Fluorescence analysis was done using the FV10 software (Olympus). Hoechst 33342 was used to stain cell nuclei. Antibodies used are indicated and Supplementary Table 3.

### Reverse transcription and real-time PCR

Total RNA was purified from whole body liver tissues at the indicated times after surgery. Tissues were grinded in liquid nitrogen, extracted using a Trizol (Ambion)/chloroform solution and centrifuged. Supernatants were precipitated with isopropanol and centrifuged again at 15.000 × g for 30 min at 4 °C. Pellets were dissolved in DEPC-treated water and the resulting solution further purified with the High Pure RNA Isolation Kit (Roche). Reverse transcription was done using random priming and the iScript Reverse Transcription Super Mix (Bio-Rad). Real-time PCR (qPCR) was performed using SYBR^®^ Select Master Mix (Life Technologies) in a Step One Thermal Cycler (Applied Biosystems) as indicated^42,43^. *Gapdh* was used to normalize target gene expression (ΔCt) and 2^−ΔΔCt^ to calculate changes in mRNA levels with respect to untreated control conditions. Primer sequences used are indicated in Supplementary Table 1.

### Chromatin Immunoprecipitation (ChIP)

Samples of whole body liver tissue (60 mg) from mice of each genotype and time point after PHx were finally minced and DNA–protein interactions stabilized by incubation in 1% formaldehyde for 15 min at room temperature with gentle agitation. Incubation was stopped by adding 0.125 M glycine for 5 min. After washing in ice-cold PBS, samples were homogenized and cell suspensions incubated for 10 min at 4 °C in SDS lysis buffer containing protease inhibitors (Complete protease inhibitor cocktail, Roche). The remaining procedures were performed as previously described^64,65^. Positive controls were done using input DNA form the same samples whereas negative controls were performed using a non-specific IgG. Real-time PCR (qPCR) was carried out using IQ-SYBR Green in a Step One Thermal Cycler (Applied Biosystems). Primers used for ChIP experiments are indicated in Supplementary Table 2.

### SDS-PAGE and immunoblotting

SDS-PAGE and immunoblotting were performed using total protein extracts essentially as described^66^. Briefly, *Ahr*^+*/*+^ and *Ahr*^*−/−*^ whole body livers were minced, homogenized in ice-cold lysis buffer (50 mM Tris–HCl pH 7.5, 150 mM NaCl, 0.5% Nonidet P-40, 1 mM phenyl-methyl sulfonyl fluoride, 1 mM NaF, 1 mM sodium orthovanadate, 1 mM DTT, 10 mM β-glycerophosphate and 4 μg/μl complete protease inhibitor cocktail (Roche)) and centrifuged at 15.000 × g for 30 min at 4 °C. Protein concentration was quantified in the supernatants using the Coomassie Plus protein assay reagent (Pierce) and bovine serum albumin as standard. Aliquots of 20–30 μg total protein were electrophoresed in 8% SDS-PAGE gels which were transferred to nitrocellulose membranes by electroblotting. Membranes were blocked in TBS-T (50 mM Tris–HCl pH 7.5, 150 mM NaCl, 0.2% Tween-20) containing 5% non-fat milk. All membranes were cut around the molecular weights between which we expect our protein of interest to be found and then blots were sequentially incubated with the corresponding primary and secondary antibodies, washed in TBS-T and revealed using the Super-signal luminol substrate (Pierce) in a ChemiDoc XRS + equipment (Bio-Rad). Antibodies used are indicated in Supplementary Table 3.

### Extraction and separation of nuclear and cytoplasmic proteins

Whole body livers from *Ahr*^+*/*+^ and *Ahr*^*−/−*^ mice were finally minced, filtered and the resulting cell suspensions washed and resuspended in PBS at 4 °C and centrifuged at 14.000 × g for 30 s at 4 °C. Samples were then resuspended in 150 µl of buffer A (PBS containing 0.5 mM DTT, 0.2 mM PMSF, 0.05% Nonidet P-40 and 4 µg/µl of complete protease inhibitor cocktail (Roche)) for 10 min at 4 °C and centrifuged under the same conditions. Supernatants were further centrifuged, and the non-precipitated fractions conserved as cytoplasmic proteins. Precipitates from this last centrifugation were resuspended in buffer A and centrifuged two more cycles to eliminate remaining cytoplasmic proteins. Final precipitates were resuspended in buffer B (PBS containing 150 mM NaCl, 0.5 mM DTT, 0.2 mM PMSF, 25% glycerol and 4 µg/µl of complete protease inhibitor cocktail (Roche)) for 30 min at 4 °C. Samples were afterwards subjected to cycles of 30 s sonication and 2 min incubation on ice until total dissolution of precipitated proteins. Next, a centrifugation step was carried out at 14.000 × g for 30 min at 4 °C and the supernatant collected as the nuclear fractions. Protein purity of the nuclear and cytosolic fractions was analyzed by immunoblotting using antibodies against the catalytic subunit of Histone 4 (Sigma-Aldrich) and Gapdh (Cell Signaling), respectively.

### Glucose measurement

Glucose concentration in blood samples from *Ahr*^+*/*+^ and *Ahr*^*−/−*^ mice was measured at each time point after PHx using the Glucocard G+ meter (Arkray Factory) following the instructions provided by the manufacturer. Mice were provided with free access to food and water.

### Hexokinase activity

Samples containing 25 mg of liver tissue from *Ahr*^+*/*+^ and *Ahr*^*−/−*^ mice were disaggregated by incubation in 0.05% (w/v) trypsin solution containing 0.53 mM EDTA for 10 min at 37 °C and centrifuged. After washing twice in PBS containing 1 mM EDTA and 10% (v/v) FBS and once in PBS, samples were resuspended in hexokinase (HK) assay buffer and homogenized through a 30 g syringe. Homogenized samples were analyzed using the pico-probe Hexokinase activity assay kit (Biovision) following manufacturer's indications.

### Analysis of TCGA data

TCGA clinical data from resected liver hepatocarcinoma (LIHC) was retrieved from the GDC portal (https://portal.gdc.cancer.gov), while paired RNA-seq normalized RPKM was downloaded from linkedomics web server (http://www.linkedomics.org/). MATLAB was used to overlap both datasets, and two distinct groups (AhR high expression and AhR low expression) were obtained by selecting the samples above or below the AhR median expression. Afterwards, a log-rank test for Kaplan–Meier survival data was applied for statistical significance, while a cumulative density function was used for plotting the results.

### Statistical analyses

Quantitative data are shown as mean ± SD. Comparison between experimental conditions was done using GraphPad Prism 6.0 software (GraphPad). The student´s t test was used to analyze differences between two experimental groups and ANOVA for the analyses of three or more groups. The Mann–Whitney non-parametric statistical method was used to compare rank variations between independent groups.

## Supplementary Information

Supplementary Tables.

Supplementary Figures.

## Data Availability

All data generated or analysed during this study are included in this published article (and its supplementary information files) and are available from the corresponding authors on reasonable request.

## Abbreviations

Ahr: Aryl hydrocarbon receptor
PH: 2/3 Partial hepatectomy
