# Supplementary material for: Liver regeneration after partial hepatectomy is improved in the absence of aryl hydrocarbon receptor
Source: Sci Rep. 2022 Sep 14;12:15446. doi: 10.1038/s41598-022-19733-0 (PMC9474532; doi:10.1038/s41598-022-19733-0)
Supplement: Supplementary file 1 — Supplementary Tables. [file 41598_2022_19733_MOESM1_ESM.pptx]

## Slide 1
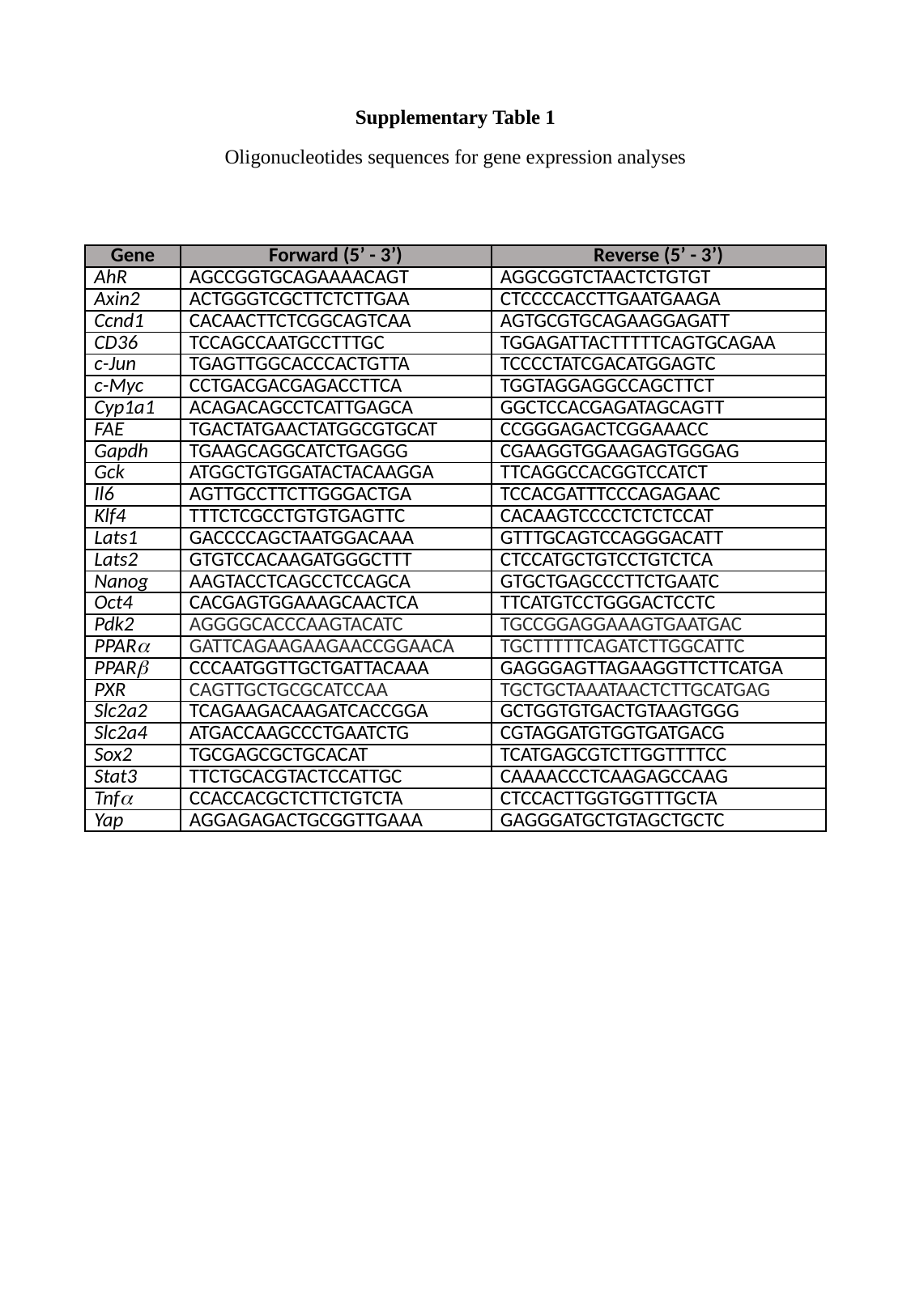

Supplementary Table 1
Oligonucleotides sequences for gene expression analyses
| Gene | Forward (5’ - 3’) | Reverse (5’ - 3’) |
| --- | --- | --- |
| AhR | AGCCGGTGCAGAAAACAGT | AGGCGGTCTAACTCTGTGT |
| Axin2 | ACTGGGTCGCTTCTCTTGAA | CTCCCCACCTTGAATGAAGA |
| Ccnd1 | CACAACTTCTCGGCAGTCAA | AGTGCGTGCAGAAGGAGATT |
| CD36 | TCCAGCCAATGCCTTTGC | TGGAGATTACTTTTTCAGTGCAGAA |
| c-Jun | TGAGTTGGCACCCACTGTTA | TCCCCTATCGACATGGAGTC |
| c-Myc | CCTGACGACGAGACCTTCA | TGGTAGGAGGCCAGCTTCT |
| Cyp1a1 | ACAGACAGCCTCATTGAGCA | GGCTCCACGAGATAGCAGTT |
| FAE | TGACTATGAACTATGGCGTGCAT | CCGGGAGACTCGGAAACC |
| Gapdh | TGAAGCAGGCATCTGAGGG | CGAAGGTGGAAGAGTGGGAG |
| Gck | ATGGCTGTGGATACTACAAGGA | TTCAGGCCACGGTCCATCT |
| Il6 | AGTTGCCTTCTTGGGACTGA | TCCACGATTTCCCAGAGAAC |
| Klf4 | TTTCTCGCCTGTGTGAGTTC | CACAAGTCCCCTCTCTCCAT |
| Lats1 | GACCCCAGCTAATGGACAAA | GTTTGCAGTCCAGGGACATT |
| Lats2 | GTGTCCACAAGATGGGCTTT | CTCCATGCTGTCCTGTCTCA |
| Nanog | AAGTACCTCAGCCTCCAGCA | GTGCTGAGCCCTTCTGAATC |
| Oct4 | CACGAGTGGAAAGCAACTCA | TTCATGTCCTGGGACTCCTC |
| Pdk2 | AGGGGCACCCAAGTACATC | TGCCGGAGGAAAGTGAATGAC |
| PPARa | GATTCAGAAGAAGAACCGGAACA | TGCTTTTTCAGATCTTGGCATTC |
| PPARb | CCCAATGGTTGCTGATTACAAA | GAGGGAGTTAGAAGGTTCTTCATGA |
| PXR | CAGTTGCTGCGCATCCAA | TGCTGCTAAATAACTCTTGCATGAG |
| Slc2a2 | TCAGAAGACAAGATCACCGGA | GCTGGTGTGACTGTAAGTGGG |
| Slc2a4 | ATGACCAAGCCCTGAATCTG | CGTAGGATGTGGTGATGACG |
| Sox2 | TGCGAGCGCTGCACAT | TCATGAGCGTCTTGGTTTTCC |
| Stat3 | TTCTGCACGTACTCCATTGC | CAAAACCCTCAAGAGCCAAG |
| Tnfa | CCACCACGCTCTTCTGTCTA | CTCCACTTGGTGGTTTGCTA |
| Yap | AGGAGAGACTGCGGTTGAAA | GAGGGATGCTGTAGCTGCTC |

## Slide 2
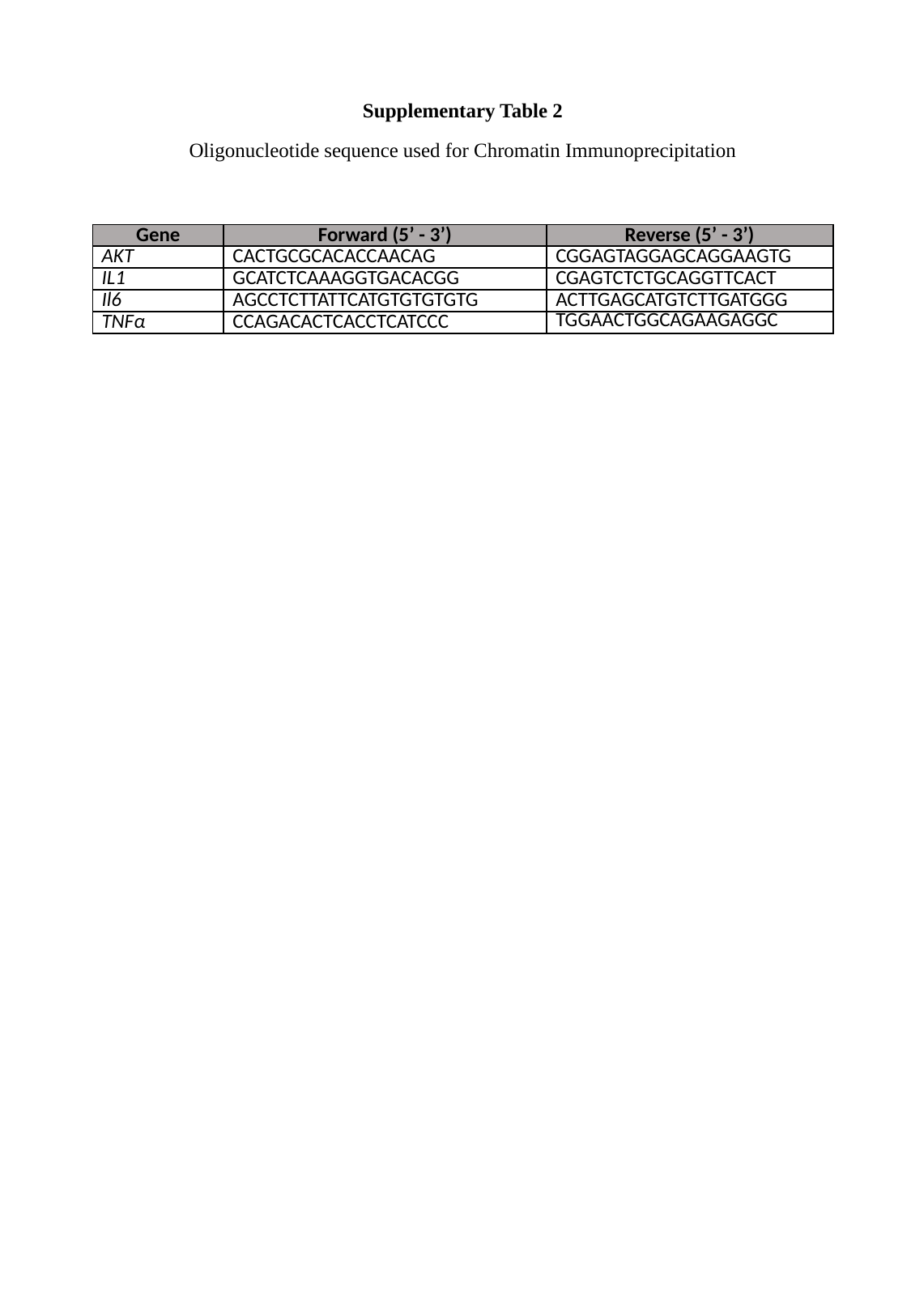

Supplementary Table 2
Oligonucleotide sequence used for Chromatin Immunoprecipitation
| Gene | Forward (5’ - 3’) | Reverse (5’ - 3’) |
| --- | --- | --- |
| AKT | CACTGCGCACACCAACAG | CGGAGTAGGAGCAGGAAGTG |
| IL1 | GCATCTCAAAGGTGACACGG | CGAGTCTCTGCAGGTTCACT |
| Il6 | AGCCTCTTATTCATGTGTGTGTG | ACTTGAGCATGTCTTGATGGG |
| TNFα | CCAGACACTCACCTCATCCC | TGGAACTGGCAGAAGAGGC |

## Slide 3
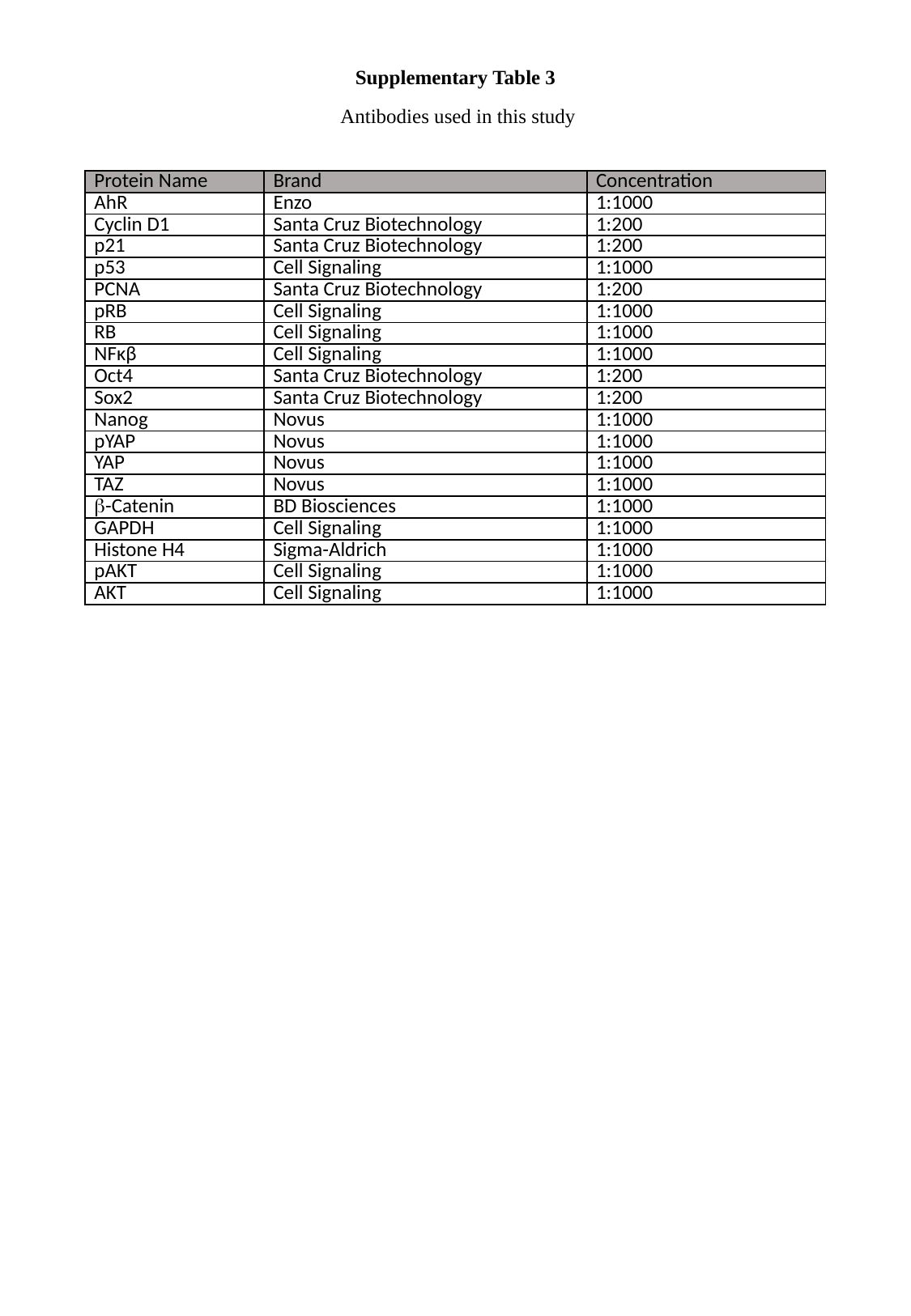

Supplementary Table 3
 Antibodies used in this study
| Protein Name | Brand | Concentration |
| --- | --- | --- |
| AhR | Enzo | 1:1000 |
| Cyclin D1 | Santa Cruz Biotechnology | 1:200 |
| p21 | Santa Cruz Biotechnology | 1:200 |
| p53 | Cell Signaling | 1:1000 |
| PCNA | Santa Cruz Biotechnology | 1:200 |
| pRB | Cell Signaling | 1:1000 |
| RB | Cell Signaling | 1:1000 |
| NFκβ | Cell Signaling | 1:1000 |
| Oct4 | Santa Cruz Biotechnology | 1:200 |
| Sox2 | Santa Cruz Biotechnology | 1:200 |
| Nanog | Novus | 1:1000 |
| pYAP | Novus | 1:1000 |
| YAP | Novus | 1:1000 |
| TAZ | Novus | 1:1000 |
| b-Catenin | BD Biosciences | 1:1000 |
| GAPDH | Cell Signaling | 1:1000 |
| Histone H4 | Sigma-Aldrich | 1:1000 |
| pAKT | Cell Signaling | 1:1000 |
| AKT | Cell Signaling | 1:1000 |
