# Supplementary material for: Liver regeneration after partial hepatectomy is improved in the absence of aryl hydrocarbon receptor
Source: Sci Rep. 2022 Sep 14;12:15446. doi: 10.1038/s41598-022-19733-0 (PMC9474532; doi:10.1038/s41598-022-19733-0)
Supplement: Supplementary file 2 — Supplementary Figures. [file 41598_2022_19733_MOESM2_ESM.pdf]

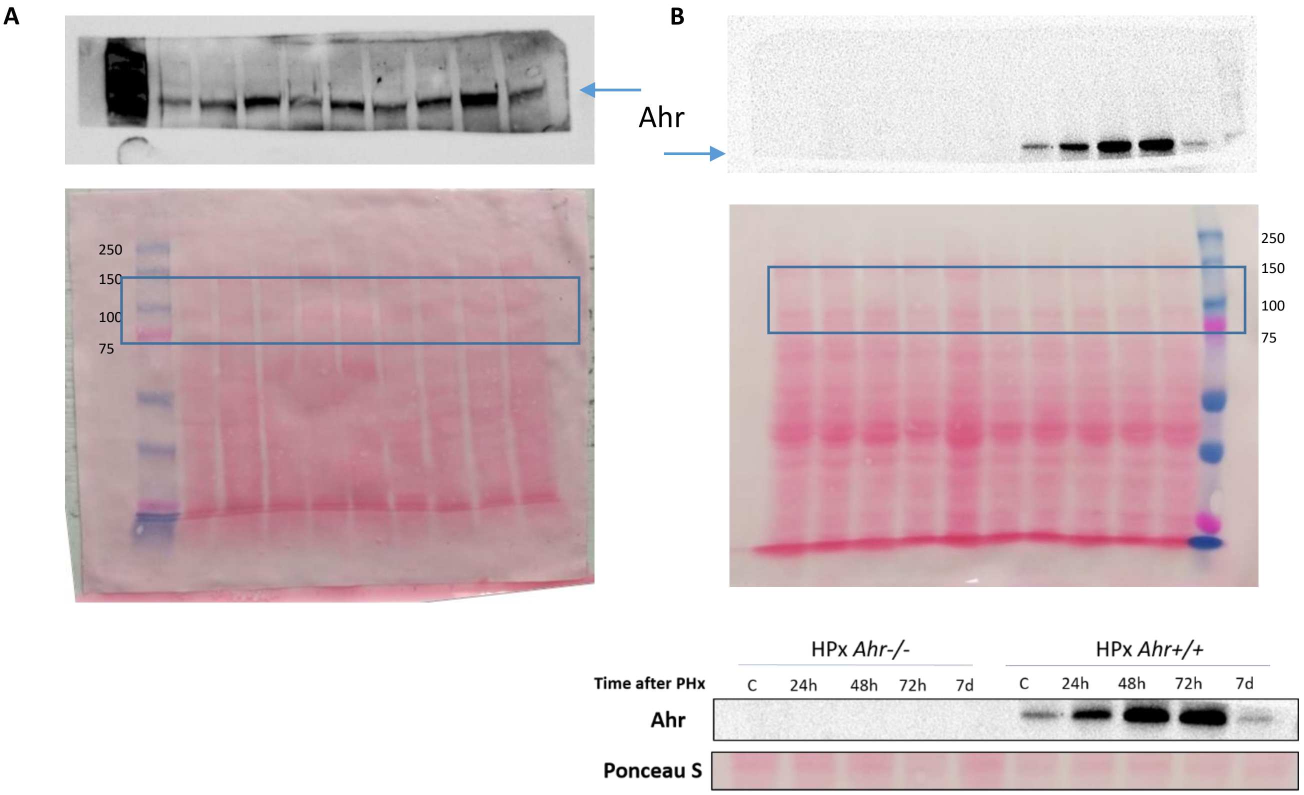

Supplementary Figure S1. Absence of Ahr protein in the liver of AhR-null mice before and after PHx. **(A)** Ahr protein expression was analyzed in liver extracts of *Ahr*$^{+/+}$ mice after PHx (originals corresponding to the figure 1A). **(B)** Ahr protein expression was analyzed in liver extracts of *Ahr*$^{+/+}$ and *Ahr*$^{-/-}$ mice at the indicated times after 2/3 PHx by immunoblotting. Ponceau staining was used to normalize protein levels. The region of the membrane incubated with the antibodies and the full-length gels showing Ponceau staining are shown. At least three mice of each genotype were analyzed.

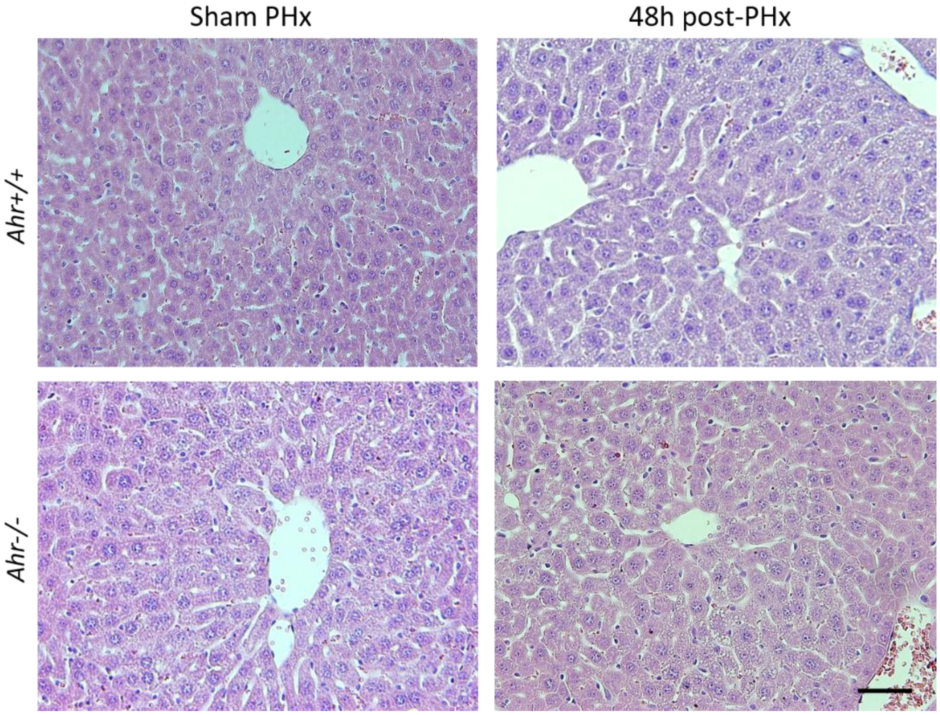

Supplementary Figure S2. Liver structure of *AhR*+/+ and *AhR*-/- mice 48 h after 2/3 PHx. Representative images of Hematoxylin and Eosin (H&E) staining are shown. Sham-operated mice are also shown as controls. Bar corresponds to 100 μm.

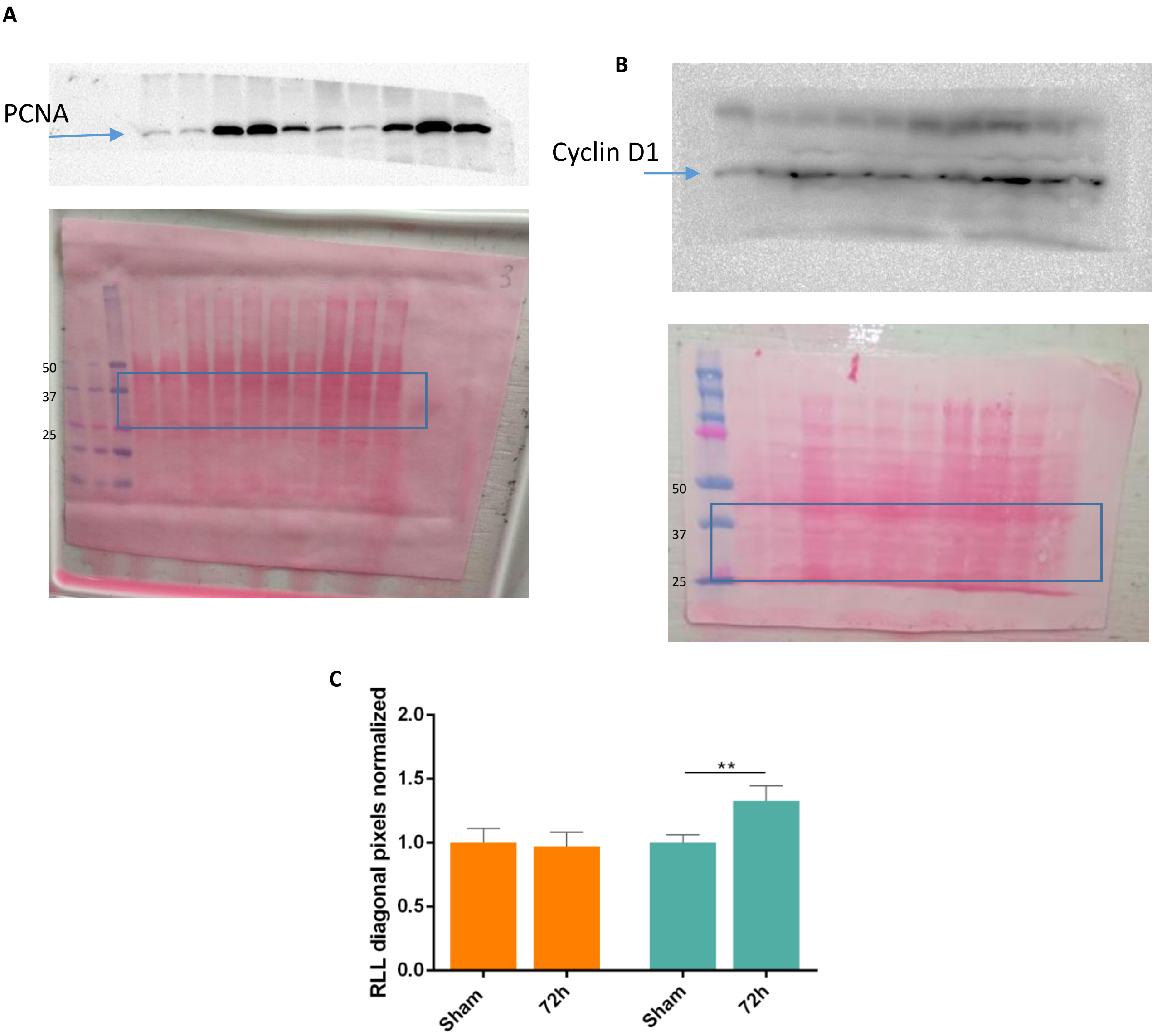

Supplementary Figure S3. Proliferation are increased in livers of *Ahr*$^{-/-}$ mice after PHx. Protein expression of (A) PCNA and (B) Cyclin D1 was determined by immunoblotting in liver extracts of *Ahr*$^{+/+}$ and *Ahr*$^{-/-}$ mice at the indicated time points after PHx (originals corresponding to the figure 2A and 2F, respectly). Ponceau staining was used to normalize protein levels. The region of the membrane incubated with the antibodies and the full-length gels showing Ponceau staining are shown. At least three mice of each genotype were analyzed. (C) Determination of the diagonal vs lateral sizes of the right lobe 72 h after PHx and in sham *Ahr*$^{+/+}$ and *Ahr*$^{-/-}$ mice.

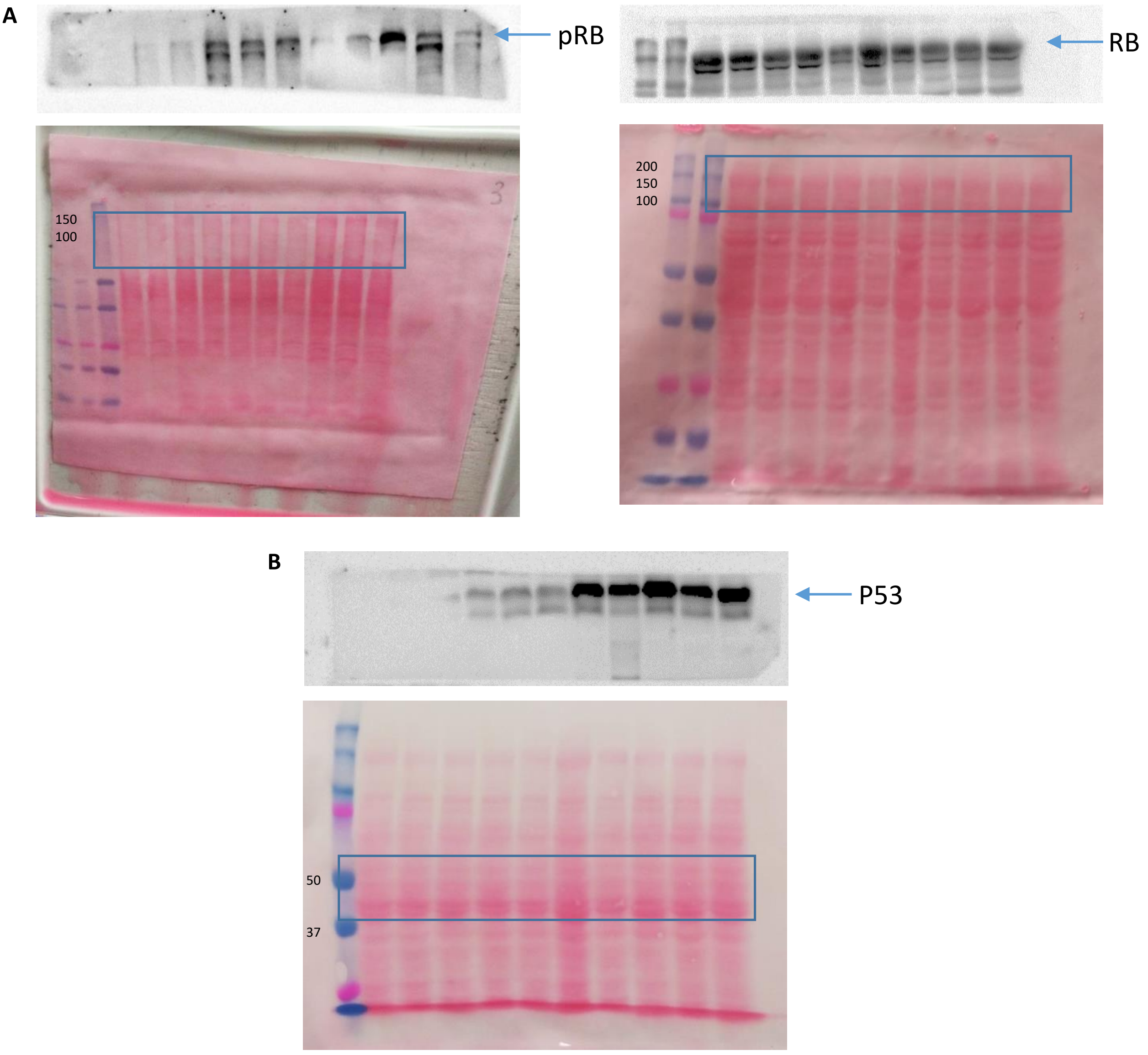

Supplementary Figure S4. Proliferation rates are increased in livers of *Ahr*$^{-/-}$ mice after PHx. Protein expression of pRb (Ser 807/811), Rb and P53 as determined by immunoblotting in liver extracts of *Ahr*$^{+/+}$ and *Ahr*$^{-/-}$ mice at the indicated time points after PHx (originals corresponding to the figure 3A and 3C, respectively). Ponceau staining was used to normalize protein levels. The region of the membrane incubated with the antibodies and the full-length gels showing Ponceau staining are shown. At least three mice of each genotype were analyzed.

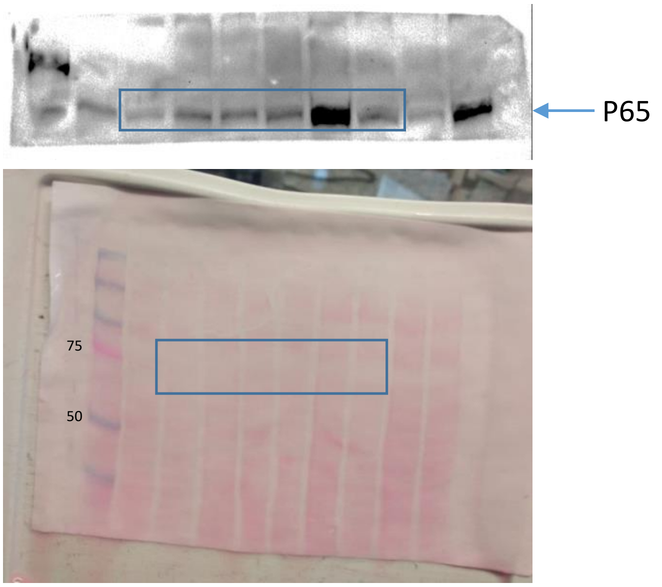

Supplementary Figure S5. The activation of NF-kB was higher in the absence of Ahr. Protein expression of p-p65-NFκβ (Ser536) was determined by immunoblotting in liver extracts of *Ahr*$^{+/+}$ and *Ahr*$^{-/-}$ mice at the indicated time points after PHx (originals corresponding to the figure 4E). Ponceau staining was used to normalize protein levels. The region of the membrane incubated with the antibodies and the full-length gels showing Ponceau staining are shown. At least three mice of each genotype were analyzed.

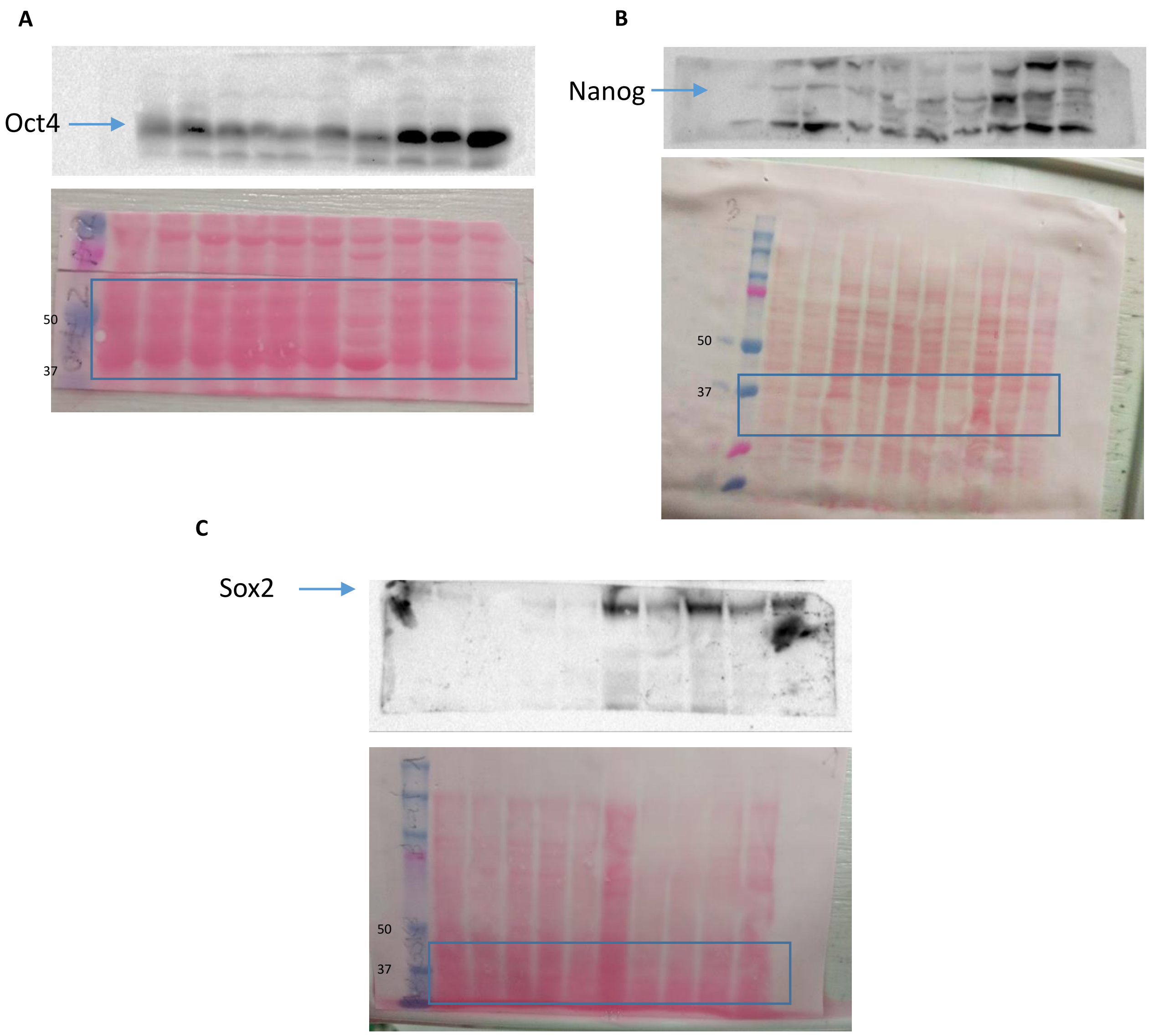

Supplementary Figure S6. Pluripotency markers are increased in *Ahr-/-* after PHx. Protein expression of (A) Oct4, (B) Nanog and (C) Sox2 was determined by immunoblotting in liver extracts of *Ahr*$^{+/+}$ and *Ahr*$^{-/-}$ mice at the indicated time points after PHx (originals corresponding to the figure 5E-G). Ponceau staining was used to normalize protein levels. The region of the membrane incubated with the antibodies and the full-length gels showing Ponceau staining are shown. At least three mice of each genotype were analyzed.

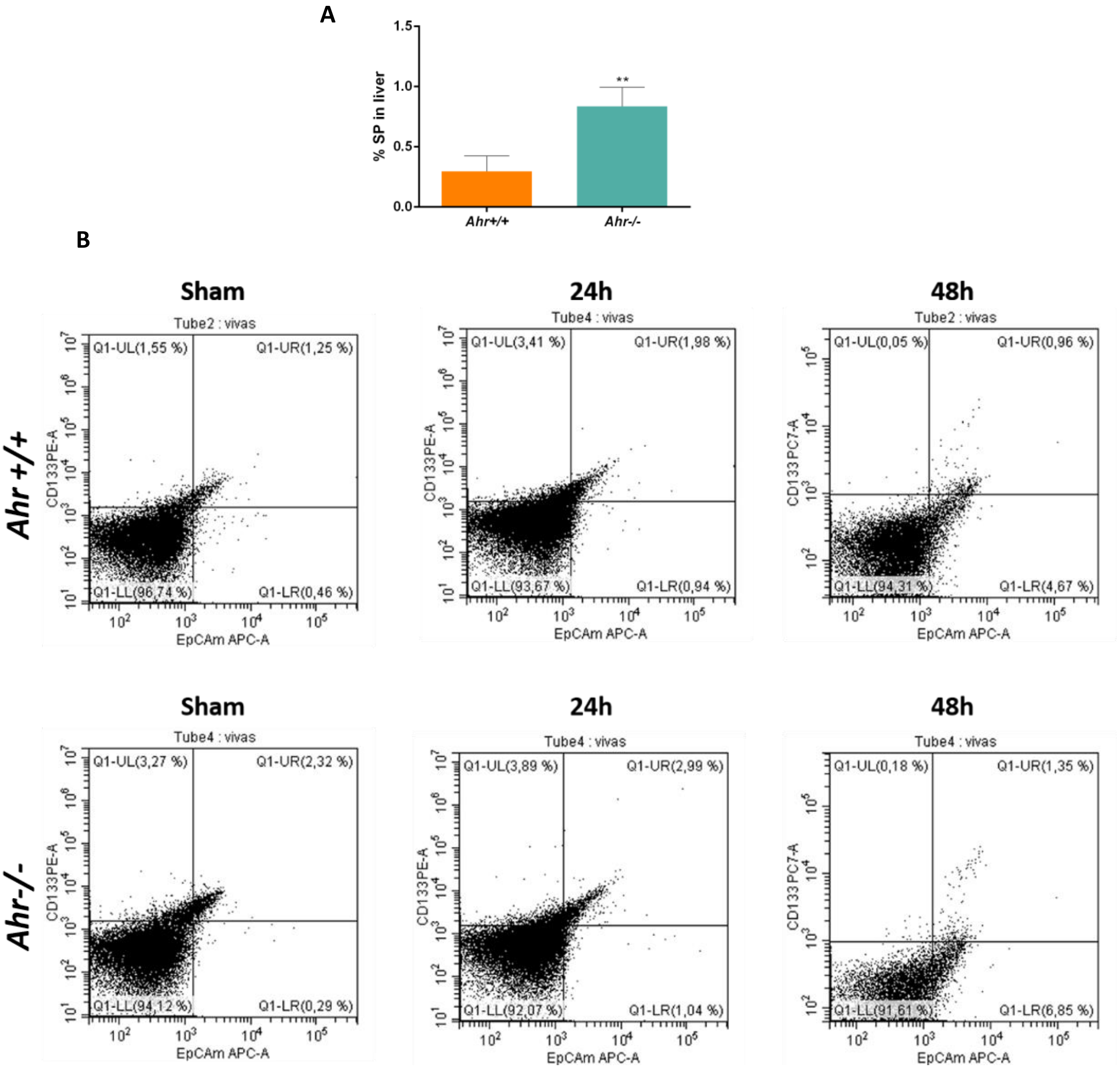

Supplementary Figure S7. Analysis of hepatic cells from *Ahr*$^{+/+}$ and *Ahr*$^{-/-}$ mice. **(A)** Quantification by flow cytometry analyses of SP cells in the liver of *Ahr*$^{+/+}$ and *Ahr*$^{-/-}$ mice under resting conditions. **(B)** Representative flow cytometry analysis of undifferentiated CD133+EpCam+ cells in livers isolated from mice of both genotypes 24 h and 48 h after 2/3 PHx. Cells obtained from sham-operated control livers were also processed. CD133+EpCam+ cells are expressed with respect to the total number of liver cells.. **p< 0.01. Data are shown as mean ± SD. Three mice of each genotype were used.

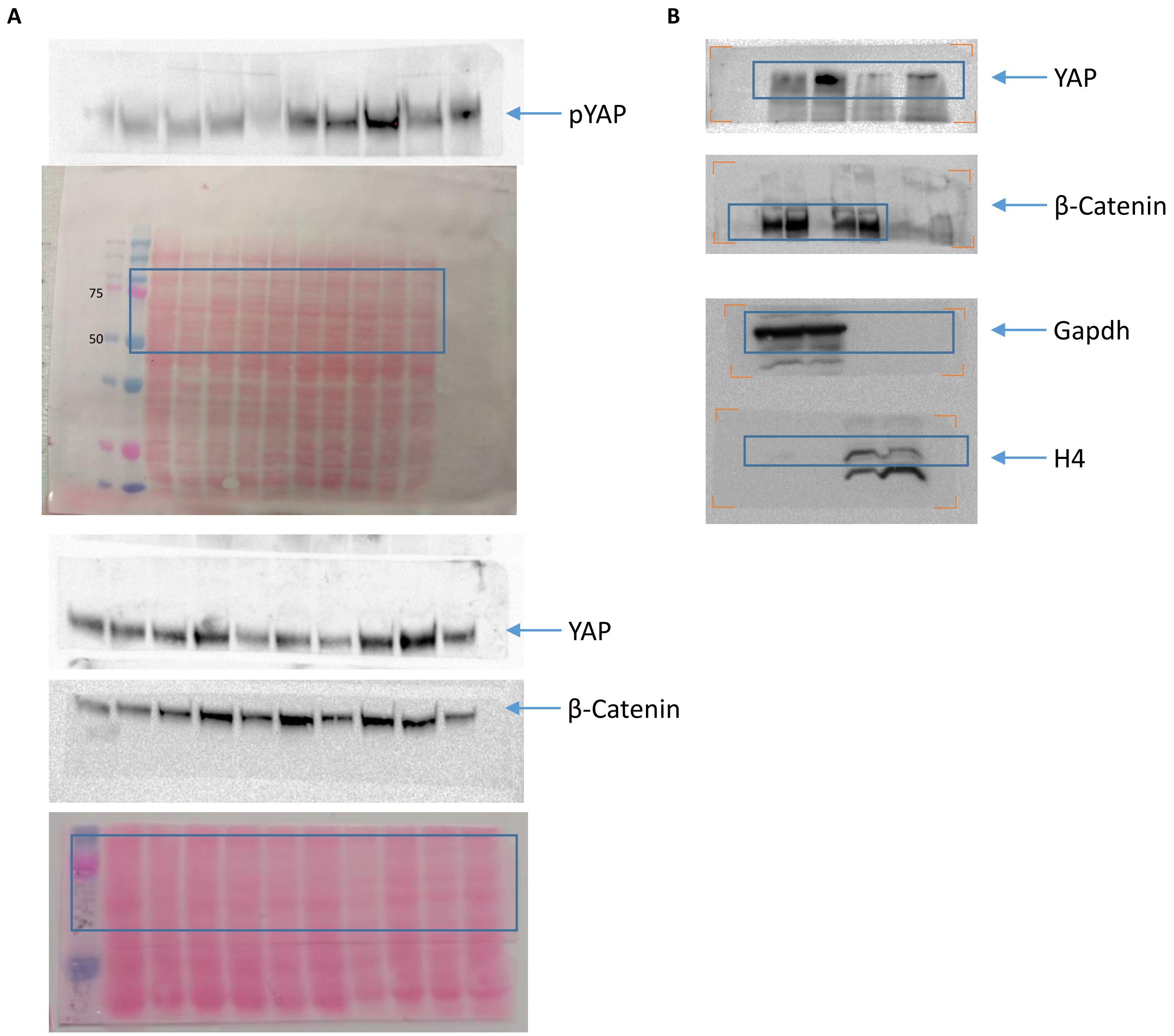

Supplementary Figure S8. Ahr regulated Hippo and Wnt signalling pathways during liver regeneration. (A) Protein expression of pYap, Yap and β-Catenin was determined by immunoblotting in liver extracts of *Ahr*$^{+/+}$ and *Ahr*$^{-/-}$ mice at the indicated time points after PHx (originals corresponding to the figure 7A-B, H). (B) Cytosolic and nuclear levels of β-Catenin and YAP were determined by immunoblotting using specific antibodies GAPDH and Histone H4 were used as controls for cytosolic and nuclear protein, respectively (originals corresponding to the figure 7D). Ponceau staining was used to normalize protein levels. The region of the membrane incubated with the antibodies and the full-length gels showing Ponceau staining are shown. At least three mice of each genotype were analyzed.

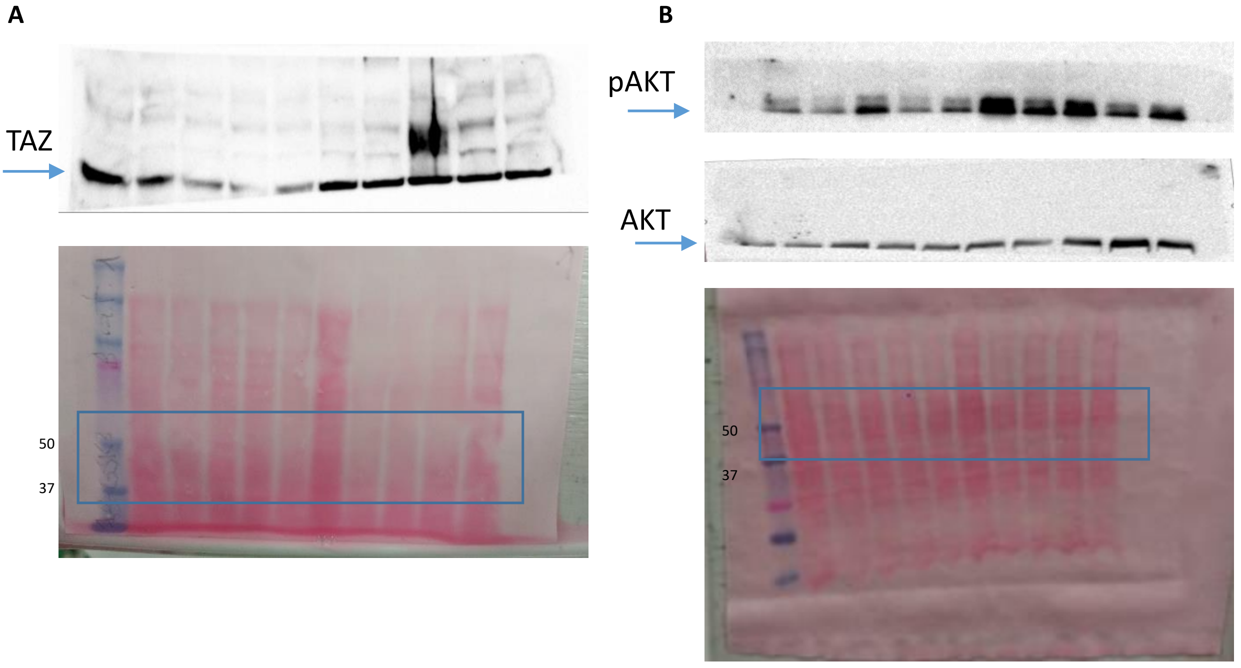

Supplementary Figure S9. Ahr regulated Hippo and Wnt signalling pathways during liver regeneration. Protein expression of (A) Taz, (B) pAKT and AKT was determined by immunoblotting in liver extracts of *Ahr*$^{+/+}$ and *Ahr*$^{-/-}$ mice at the indicated time points after PHx (originals corresponding to the figure 7E and 8L, respectively). Ponceau staining was used to normalize protein levels. The region of the membrane incubated with the antibodies and the full-length gels showing Ponceau staining are shown. At least three mice of each genotype were analyzed.
